# Catalytic Enantioselective Addition of Alkylzirconium Reagents to Aliphatic Aldehydes

**DOI:** 10.3390/molecules26154471

**Published:** 2021-07-24

**Authors:** Jade Vaccari, María José González-Soria, Nicholas Carter, Beatriz Maciá

**Affiliations:** Division of Chemistry & Environmental Science, Manchester Metropolitan University, Oxford Road, Manchester M1 5GD, UK; JADE.VACCARI@stu.mmu.ac.uk (J.V.); mariajo.gonzalez.s@gmail.com (M.J.G.-S.); ncarter1990@gmail.com (N.C.)

**Keywords:** alkenes, Schwartz reagent, enantioselective catalysis, titanium-diol complex, aliphatic aldehydes

## Abstract

A catalytic methodology for the enantioselective addition of alkylzirconium reagents to aliphatic aldehydes is reported here. The versatile and readily accessible chiral Ph-BINMOL ligand, in the presence of Ti(O*^i^*Pr)_4_ and a zinc salt, facilitates the reaction, which proceeds under mild conditions and is compatible with functionalized nucleophiles. The alkylzirconium reagents are conveniently generated in situ by hydrozirconation of alkenes with the Schwartz reagent. This work is a continuation of our previous work on aromatic aldehydes.

## 1. Introduction

Chiral aliphatic secondary alcohols—ubiquitous (sub)structures in natural products—play a very important role in chemical communication among living organisms, serving as sex, aggregation, alarm and trail pheromones, attractants or repellents. Chiral aliphatic alcohols are very valuable organic materials with applications from pharmaceutical and agricultural to food additives, fragrances and cosmetics [1].

The enantioselective synthesis of aliphatic alcohols has been recognized as a long-term interest in the chemistry community, and a wide variety of catalytic asymmetric methodologies [2,3] allow access to these relevant moieties in high enantiomeric excess, including reduction or hydrogenation of prochiral aliphatic ketones [4,5] and addition of alkyl organometallic reagents to aliphatic aldehydes [6,7]. The addition of alkylzinc [8,9,10,11], alkylaluminum [12,13,14], alkyltitanium [15,16,17] and, more recently, alkyl Grignard reagents [18,19,20,21,22,23,24,25,26] to aliphatic aldehydes has been extensively studied. However, the implementation of these methodologies in industrial processes and large scale reactions, is often hampered by the high reactivity, and sometimes pyrophoric character, of these premade, non-stabilized organometallic nucleophiles [27]. The use of less reactive nucleophiles, such as organozirconium compounds, circumvents some of the negative implications associated with these premade organometallic reagents; such as the need for cryogenic temperatures (needed to obtain high levels of enantioselectivity) and the incompatibilities with several functional groups [28]. In addition, organozirconium reagents are readily accessible via (in-situ) hydrozirconation of alkenes using the Schwartz reagent (Cp_2_ZrHCl) [29,30,31], and alkenes are classed as inexpensive, abundant and easy to handle precursors [32].

Organozirconium reagents [33,34,35] are relatively inert toward carbonyl compounds [36], but their nucleophilic character can be enabled by different catalysts or stoichiometric mediators [37,38,39,40,41,42]. Thus, the addition of organozirconium reagents to aldehydes [43,44,45,46,47,48,49], ketones [50,51], enones [52,53,54], epoxides [55] and isocyanates [56] is possible in the presence of Ag(I), ZnBr_2_ or Me_2_Zn; although these protocols are rarely enantioselective [57,58,59,60,61,62,63]. The addition of alkenylzirconocenes to aldehydes and ketones, reported by Wipf [45,64,65] and Walsh [66], respectively; together with our recent work on the addition of alkylzirconium reagents to aromatic aldehydes [67], are, to the best of our knowledge, the only catalytic asymmetric methodologies for the addition of organozirconium reagents to carbonyl compounds.

Here, we report the enantioselective addition of alkylzirconium reagents to aliphatic aldehydes, using a chiral titanium-(Ph-BINMOL) complex as catalyst in the presence of a zinc salt, under industrially relevant reaction conditions. The in-situ preparation of the alkylzirconium nucleophile allows the synthesis of chiral secondary aliphatic alcohols bearing functional groups that are not compatible with other premade organometallic reagents. This work is an extension of our previous, recently published work [67], where only aromatic aldehydes were employed as substrates.

## 2. Results and Discussion

The large number of aliphatic secondary alcohols in natural products and pharmaceutical compounds makes enantioselective routes to these materials important [68]. The catalytic enantioselective 1,2-addition reaction of alkyl carbon nucleophiles to aliphatic aldehydes is one of the most efficient and straightforward strategies to chiral aliphatic alcohols. This approach, however, is often challenging, due to the highly enolizable character of aliphatic aldehydes, together with their multiple conformations and lack of π-stacking interactions with the catalysts.

Our investigations started by assessing the use of (*R_a_*,*S*)-Ph-BINMOL ligand **L1** [69,70,71] in the addition of 1-hexene (**2a**) to cyclohexanecarboxaldehyde (**1a**, Table 1). The corresponding alkylzirconium reagent was prepared by treatment of 1-hexene (**2a**, 2.2 eq) with 2.0 eq. of Schwartz reagent (Cp_2_ZrHCl); a change from a white suspension to a clear yellow solution suggested that the nucleophile had successfully formed [29,30,31]. This was then added to a solution of cyclohexanecarboxaldehyde (1.0 eq., 0.125 M), Ti(O*^i^*Pr)_4_ (1.5 eq) and Ph-BINMOL (**L1**, 20 mol %) in DCM at 35 °C (Table 1, entry 1), following known procedures for the enantioselective addition of different organometallic reagents to carbonyl compounds using Ph-BINMOL ligands [72,73,74,75,76,77,78]. Unfortunately, under these reaction conditions, only the alcohol **4a** was obtained (Table 1, entry 1), presumably formed by the reduction of aldehyde **1a** by the metal-bonded hydride produced after a β-hydride elimination process in the organozirconium reagent.

Our previous work on the addition of organozirconium reagents to aromatic aldehydes [67] demonstrates that the use of a zinc salt as an additive facilitates the enantioselective nucleophilic addition. Under the initial reaction conditions, different amounts (from 0.025 to 1.5 eq) of ZnBr_2_ were tested (Table 1, entries 2–5). While the use of 0.025 (entry 2) and 1.0 eq (entry 4) of ZnBr_2_ provided the reduced product **4a** exclusively, the reaction proceeded well with 0.5 eq of ZnBr_2_ (entry 3), providing the desired chiral secondary alcohol **3aa** in 99% conversion and 86% ee. The use of 1.5 eq of ZnBr_2_ afforded **3aa** in slightly lower conversion and enantioselectivity (95% conv and 18% ee, entry 5). Next, we performed a screening of different amounts of titanium isopropoxide (entries 6–9). Increasing the Ti(O*^i^*Pr)_4_ loading from 1.5 to 2 eq., resulted in 75% conversion to the reduced product **4a**, as well as lower enantioselectivity for the desired product **3aa** (Table 1, entry 6). Lowering the equivalents of Ti(O*^i^*Pr)_4_ to 1 eq. resulted in >99% conversion to the reduced product **4a** (Table 1, entry 7). Varying the ratios between Ti(O*^i^*Pr)_4_ and ZnBr_2_ to 2:1 and 2:2 (Table 1, entries 8 and 9) showed no improvement in enantioselectivity or conversion when compared with the original ratio of 3:1 (Table 1, entry 3).

Having found the optimal ratio between the zinc additive and the titanium source, we explored the reaction at room temperature, in the hope of increasing the enantioselectivity and making the reaction more sustainable. Unfortunately, the conversion to the by-product **4a** substantially increased under these conditions (Table 1, entry 10).

The use of CuCl as additive, instead of ZnBr_2_, was also evaluated (Table 1, entries 11 and 12), but provided slightly lower enantioselectivity at both 35 °C and room temperature (64 and 40% ee, respectively) than the zinc salt.

We have previously reported that 4-Py-BINMOL (**L2**) is the most effective ligand for the catalytic enantioselective 1,2-addition of Grignard reagents to aliphatic aldehydes [26]. However, when **L2** was employed as the ligand for the addition of 1-hexene (**2a**) to cyclohexanecarboxaldehyde (**1a**), only 32% conversion was observed for the desired **3aa** with lower ee (8%), while the formation of the by-product **4a** substantially increased (Table 1, entry 13).

**Scheme 1:**
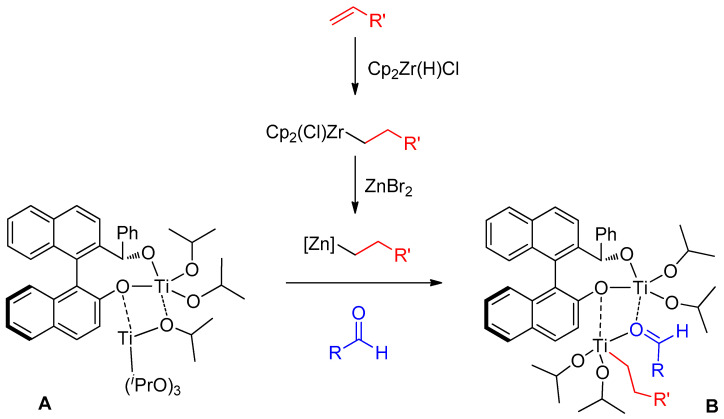
Proposed titanium-**L1** complex (**A**) and proposed active intermediate (**B**) containing both aldehyde (RCOH) and nucleophile (CH_2_CH_2_R’).

There are multiple mechanistic pathways in which this catalytic enantioselective 1,2-addition reaction of organozirconium reagents could proceed. We hypothesize that the in situ generated organozirconium reagents undergo transmetallation with the zinc bromide, followed by a second transmetallation with the excess of titanium isopropoxide to provide catalytic intermediate/species similar to those proposed by Seebach and Walsh on the Ti(O*^i^*Pr)_4_ assisted addition of organozinc reagents to aldehydes (Scheme 1) [15,16]. It cannot be ignored, however, that the activation of aldehydes via a zinc-halide complexation is a well-known effect [79]. It is worth noting that control reactions performed in the absence of Ti(O*^i^*Pr)_4_ and (*R*_a_,*S*)-Ph-BINMOL (**L1**) resulted in no conversion to our desired product **3aa** but 11% and >99% conversion to the reduced product **4a**, respectively (Table 1, entries 14 and 15).

With the now optimised conditions [2.2 eq. of alkene, 2 eq. of Cp_2_ZrCl, 1.5 eq. of Ti(O*^i^*Pr)_4_, 0.5 eq. of ZnBr_2_, 20 mol % of **L1**, at 35 °C in DCM; Table 1, entry 3], the scope of the reaction with different aliphatic aldehydes was investigated (Table 2). The addition of 1-hexene (**2a**) to isobutyraldehyde (**1b**) and 2-ethylbutanal (**1c**) afforded the corresponding products **3ba** and **3ca** with excellent conversions (>99%) and enantioselectivities of 76 and 70%, respectively (entries 1 and 2). The isolated yields (42% and 54%, respectively) were moderate, due to the high volatility of the products, but the reduced by-products **4b** and **4c** were not observed in any case. Similar results were obtained for the reaction with pivaldehyde (**1d**), which afforded **3da** with 93% conversion (7% reduced product), moderate yield (50%, due to volatility of the product) and good enantioselectivity of 84% (entry 3). The use of octanal (**1e**) as the substrate resulted in excellent conversion to the desired product **3ea** (91%, along with 4% conversion to the reduced product **3e**), good enantioselectivity (74%, determined on the corresponding benzoate) and 48% isolated yield (entry 4). The addition of 1-hexene (**2a**) to both 3-phenylpropionaldehyde (**1f**) and cinnamaldehyde (**1g**) led to the desired products **3fa** and **3ga** with 40% and 35% isolated yields, respectively, and high enantiomeric excesses (74% and 78%, entries 5 and 6). The use of phenylpropargyl aldehyde (**1h**) as substrate, provided **3ha** in moderate isolated yield (40%) and moderate enantioselectivity (56%, entry 7). The configuration of alcohols **3** was assigned as (*R*) after comparison with the optical rotation values in the literature for the known compounds. For unknown compounds, where comparison was not possible, the (*R*) configuration was assumed by analogy.

Next, we evaluated the use of a variety of alkenes as nucleophiles (Table 3). The reaction with cyclohexanecarboxaldehyde (**1a**) and 4-phenyl-1-butene (**2b**) provided moderate yield (33%) and 68% enantioselectivity (entry 1). We were pleased to observe that the methodology is also compatible with functionalised alkenes (entries 2–4). The use of 4–[(*tert*-butyldimethylsilyl)oxy]-1-butene (**2c**) as a nucleophile, afforded **3ac** with 27% isolated yield and 58% enantioselectivity (entry 2). The reactions with 4-halo-1-butenes **2d** and **2e** as nucleophiles, provided the desired alcohols **3ad** and **3ae** in 51 and 36% yield, and 84 and 60% ee (determined on the corresponding benzoates), respectively. It is worth noting that the majority of the yields are low to moderate as a result of the secondary alkyl alcohols being volatile. Alkenes bearing a nitrile or a thioester group (pent-4-enenitrile and but-3-en-1-yl(phenyl)sulfane, respectively) did not provide any conversion under these reaction conditions. It is worth noting that, although the alkene scope is a bit narrow, it includes halogenated alkenes. The presence of halogens is not compatible with premade organometallic reagents [6,7]; so this is a clear advantage of this methodology. Alkenes bearing a protected alcohol are also suitable with our methodology; their analogous premade organometallic reagents would also be of difficult access [18].

## 3. Conclusions

In conclusion, we have successfully developed an enantioselective 1,2-addition of alkylzirconium reagents to aliphatic aldehydes, using catalytic amounts of the very versatile (*R_a_*,*S*)-Ph-BINMOL ligand **L1**, in the presence of titanium isopropoxide and zinc bromide as additives. The alkylzirconium nucleophiles are generated in situ by hydrozirconation of alkenes with Schwartz reagent, thus avoiding the use of premade organometallic reagents. The one-pot reaction proceeds under mild conditions, with enantioselectivities in the range 56–86%, good to high conversions (53–99%) and moderate yields (27–54%). This methodology allows the synthesis of very valuable chiral secondary alcohols, difficult to access by the addition of classical premade organometallic reagents to carbonyls. In addition, the scope of the reaction includes a range of functionalised nucleophiles.

## 4. Materials and Methods

General Considerations: ^1^H-NMR, ^13^C-NMR, and ^31^P-NMR spectra were recorded on a JEOL^®^ ECS-400 NMR spectrometer (400, 100.6 and 162 MHz, respectively) using CDCl_3_ as solvent. The chemical shift values were recorded in ppm with the residual CDCl_3_ referenced to 7.26 and 77.00 for ^1^H NMR and ^13^C NMR respectively. Data is reported as follows: chemical shift, multiplicity (singlet = s, doublet = d, triplet = t, quartet = q, multiplet = m), coupling constants (*J*) in Hz and integration. Infrared Spectra were recorded on a Nicolet^®^ 380 Fourier Transform Infrared Spectrometer and only the most significant frequencies have been reported (in cm^−1^) for characterisation. Optical rotation measurements were performed on Bellingham + Stanley^®^ ADP220 Polarimeter with a 0.5 cm cell (*c* given in g/100 mL) using DCM as a solvent. Melting point measurements were performed on Stuart^®^ SMP10 melting point apparatus and were not corrected. Conversion and low resolution mass spectra were recorded on either Agilent 6850 Series connected to an Agilent 5973 mass selective detector using a HP-5ms (30 m × 0.25 mm × 0.25 μm) or on an Agilent Technologies^®^ 7890B GC connected to an Agilent Technologies^®^ 5977b MSD using a HP-5MS (30 m × 0.25 mm × 0.25 μm). Helium was used as the carrier gas at 10 psi, and the samples were ionized by an electronic impact (EI) source at 70 ev. Gas chromatography analysis was performed on an Agilent Technologies^®^ 7890A GC System and a Hewlett Packard^®^ 5890 Series II GC System, with a CycloSil-β (Agilent Technologies, 30 m × 0.25 mm) and a CP-Chirasil-DEX CB (Varian, 25 m × 0.25 mm) column, respectively; injector and detector temperatures: 250 °C. HPLC analysis was carried out on an Agilent 1100 series HPLC equipped with a G1313B diode array detector and a G1311A Quat pump, using the chiral column Lux 5µ Cellulose-1. High resolution mass spectra were obtained on a 6540 LC-QToF spectrometer and the samples were ionized with ESI techniques and introduced through a high pressure liquid chromatography (HPLC) using an Agilent Technologies^®^ 1260 Infinity Quaternary LC system, an Agilent 7200 Accurate Mass Q-ToF GC-MS system or a Waters Xevo G2-S, where the samples were ionized usingASAP techniques. Thin layer chromatography (TLC) was performed on Sigma Aldrich silica gel 60 F_254_ aluminium plates and visualised by UV light and/or by a staining solution of phosphomolybdic acid. Purification by column chromatography was performed using Geduran^®^ Silica gel 60 in a Biotage^®^ Isolera^TM^ System, using the eluents mentioned below. All reactions were carried out under inert conditions, using flame dried glassware and argon as the inert gas. All commercially available reagents were purchased from Acros, Alfa Aesar, Manchester Organics, Fisher, Fluorochem and Sigma-Aldrich and were used without further purification, except for all liquid aldehydes, which were freshly distilled before use. Anhydrous, THF, DCM, Et_2_O and toluene were obtained from Pure Solv^TM^ Solvent Purification Systems. Ligands (*Ra*,*S*)-Ar-BINMOLs **L1** and **L2** were prepared according to literature procedures [44] from (*R*)-BINOL, purchased from Manchester Organics. Racemic alcohols **3aa–3fa** (Table 1) were synthesised from the addition of hexylmagnesium bromide to the corresponding aldehyde. Racemic alcohols **3ab–3ae** (Table 2) were prepared using the general procedure below for the catalytic enantioselective 1,2-addition of alkenes to aliphatic aldehydes using racemic **L1** as ligand. 4–[(*tert*-Butyldimethylsilyl)oxy]-1-butene (**2c**) was prepared according to literature [80]. Spectroscopic data for new compounds and chiral GC and HPLC chromatograms are available as Supplementary Material.

General procedure for the catalytic enantioselective 1,2-addition of alkenes to aliphatic aldehydes: To a stirred suspension of Cp_2_ZrHCl (154 mg, 0.6 mmol, 2 eq.) in dry DCM (0.3 mL) under argon at RT, the corresponding alkene (**2a–e**, 0.66 mmol, 2.2 eq.) was added dropwise and the solution was stirred for 30 min. The mixture turned to a clear yellow solution, indicating the successful formation of the alkylzirconium reagent. Next, flame dried ZnBr_2_ (34 mg, 0.15 mmol, 0.5 eq.) was added to the solution at RT and stirred for 2 min. Next, a solution of Ti(O*^i^*Pr)_4_ (134 μL, 0.45 mmol, 1.5 eq.) and (*Ra*,*S*)-Ph-BINMOL (**L1**, 23 mg, 20 mol %) in dry DCM (0.1 mL) was added and stirred for an additional 2 min at RT. Finally, the freshly distilled aldehyde (**1a–h**, 0.3 mmol) was added and the solution was stirred at 35 °C for 12 h. The reaction was quenched with water (2 mL) and the layers were separated. The aqueous layer was extracted with Et_2_O (3 × 10 mL) and the combined organic layers were dried with MgSO_4_, filtered and concentrated under reduced pressure. The corresponding products were purified by flash silica gel chromatography.

(*R*)-1-cyclohexylheptan-1-ol **(3aa):** [81] Obtained as a yellow oil after purification by column chromatography (Et_2_O/hexane 3:7). 60% yield, 86% *ee*. [α]_D_^23^ = +17.4 (*c* 1.5, CH_2_Cl_2_). {^Lit^ [α]_D_^25^ = −10.5 (*c* 0.2, CHCl_3_) for 84% *ee* of *S* enantiomer}. ^1^H NMR (400 MHz, CDCl_3_) δ: 3.39–3.29 (m, 1H), 1.81–1.70 (m, 3H), 1.70–1.60 (m, 2H), 1.51–1.42 (m, 2H), 1.36–0.93 (m, 15H), 0.87 (t, *J* = 6.8 Hz, 3H). ^13^C NMR (101 MHz, CDCl_3_) δ: 76.1, 43.8, 34.0, 33.3, 33.0, 29.4, 27.8, 26.6, 26.5, 26.3, 24.8. *m*/*z*: 180 (M^+^-H_2_O, 11), 115 (26), 114 (14), 113 (47), 97 (76), 96 (21), 95 (100), 83 (10), 82 (14), 81 (10), 69 (14), 67 (16), 57 (12), 55 (59). HRMS (ASAP): *m*/*z* calculated for C_13_H_25_O [M-H]^+^: 197.1905, found: 197.1904. *ee* determination by chiral GC analysis, CP Chirasil-DEX CB column, T = 95 °C retention times: t_r_ = 42.1 min, t_r_ = 42.8 min (major enantiomer).

(*R*)-2-methylnonan-3-ol **(3ba):** [82] Obtained as a yellow oil after purification by column chromatography (Et_2_O/hexane 3:7). 42% yield, 76% *ee*. [α]_D_^23^ = +13.3 (*c* 0.6, CH_2_Cl_2_). {^Lit^ [α]_D_^25^ = −14.1 (*c* 0.7, CHCl_3_) for 96% *ee* of *S* enantiomer}. ^1^H NMR (400 MHz, CDCl_3_) δ: 3.41–3.30 (m, 1H), 1.64 (m, 1H), 1.50–1.41 (m, 2H), 1.41–1.22 (m, 9H), 0.93–0.82 (m, 9H). ^13^C NMR (101 MHz, CDCl_3_) δ: 76.9, 34.3, 33.6, 32.0, 29.6, 26.2, 22.8, 19.0, 17.2, 14.3. *m*/*z*: 140 (M^+^-H_2_O, 11), 115 (26), 97 (82), 73 (47), 69 (20), 57 (14), 55 (100). HRMS (+ESI): *m*/*z* calculated for C_10_H_21_O [M-H]^+^: 157.1592, found: 157.1587. *ee* determination by chiral GC analysis, CP Chirasil-DEX CB column, T = 120 °C, retention times: t_r_(*S*) = 43.3 min, t_r_(*R*) = 42.5 min (major enantiomer).

**(***R*)-3-ethylnonan-4-ol **(3ca):** Obtained as a yellow oil after purification by column chromatography (Et_2_O/hexane 2:8). 54% yield, 70% *ee*. [α]_D_^23^ = +6.7 (*c* 0.4, CH_2_Cl_2_). FTIR (neat) V_max_: 3373, 2958, 2925, 2873, 2858, 1461, 1379, 1143 cm^−1^. ^1^H NMR (400 MHz, CDCl_3_) δ: 3.69–3.52 (m, 1H), 1.45–1.16 (m, 16H), 1.06–0.81 (m, 9H). ^13^C NMR (101 MHz, CDCl_3_) δ: 73.3, 46.9, 34.2, 32.0, 29.6, 26.4, 22.8, 22.2, 21.3, 14.3, 12.1, 12.0. *m*/*z*: 168 (M^+^-H_2_O, 1), 115 (28), 101 (23), 97 (98), 83 (15), 70 (15), 69 (21), 59 (24), 57 (19), 55 (100). HRMS (+ESI): *m*/*z* calculated for C_12_H_25_O [M-H]^+^: 185.1905, found: 185.1909. *ee* determination by chiral GC analysis, CP Chirasil-DEX CB column, T = 95 °C, retention times: t_r_(*S*) = 75.1 min, t_r_(*R*) = 75.2 min (major enantiomer).

(*R*)-2,2-dimethylnonan-3-ol **(3da):** [83] Obtained as a yellow oil after purification by column chromatography (Et_2_O/cyclohexane 3:7). 50% yield, 84% *ee*. [α]_D_^23^ = +8.9 (*c* 0.9, CH_2_Cl_2_). {^Lit^ [α]_D_^25^ = +15.0 (*c* 5.1, benzene) for 84% *ee*}. FTIR (neat) V_max_: 3392, 2954, 2925, 2859, 1479, 1466, 1393, 1364, 1075, 1009, 957, 734, 703, 566, 543 cm^−1^. ^1^H NMR (400 MHz, CDCl_3_) δ: 3.17 (d, *J* = 7.4 Hz, 1H), 1.64–1.40 (m, 3H), 1.36–1.18 (m, 8H), 0.87 (br s, 12H). ^13^C NMR (101 MHz, CDCl_3_) δ: 80.1, 35.1, 32.1, 31.6, 29.6, 27.2, 25.8, 22.8, 14.3. *m*/*z*: 154 (M^+^-H_2_O, 0.18), 115 (31), 114 (13), 97 (100), 87 (28), 69 (31), 57 (40), 56 (12), 55 (80). HRMS (+ESI): *m*/*z* calculated for C_11_H_23_O [M-H]^+^: 171.1749, found: 171.1743. *ee* determination by chiral GC analysis, CP Chirasil-DEX CB column, T = 100 °C, retention times: t_r_(*S*) = 38.6 min, t_r_(*R*) = 40.2 min (major enantiomer).

(*R*)-tetradecan-7-ol **(3ea):** Obtained as a white solid after purification by column chromatography (Et_2_O/cyclohexane 3:7). 48% yield, 74% *ee* (determined on the corresponding benzoate **3ea’**). [α]_D_^22^ = +13.3 (c 0.7, CH_2_Cl_2_). M_p_ = 36–39 °C. FTIR (neat) V_max_: 3343, 2956, 2929, 2872, 1470, 1381, 1045, 952, 817 cm^−1^. ^1^H NMR (400 MHz, CDCl_3_) δ: 3.55 (s, 1H), 1.40 (br s, 7H), 1.26 (br s, 16H), 0.86 (t, *J* = 6.1 Hz, 6H). ^13^C NMR (101 MHz, CDCl_3_) δ: 72.2, 37.6, 32.0, 30.0, 30.0, 29.5, 27.0, 25.8, 25.8, 22.9, 22.8, 14.3. *m*/*z*: 196 (M^+^-H_2_O, 8), 129 (36), 115 (38), 111 (41), 97 (100), 83 (11), 69 (94), 57 (20), 55 (74). HRMS (+ESI): *m*/*z* calculated for C_14_H_29_O [M-H]^+^: 213.2219, found: 213.2213.

(*R*)-1-phenylnonan-3-ol **(3fa):** [84] Obtained as a yellow oil after purification by column chromatography (Et_2_O/cyclohexane 3:7). 40% yield, 74% *ee*. [α]_D_^26^ = −10.5 (*c* 3.8, CH_2_Cl_2_). {^Lit^ [α]_D_^21^ = −8.2 (*c* 0.3, CHCl_3_) for 72% *ee*}. ^1^H NMR (400 MHz, CDCl_3_) δ: 7.26–7.07 (m, 5H), 3.65–3.49 (m, 1H), 2.78–2.56 (m, 2H), 1.78–1.61 (m, 2H), 1.51 (s, 1H), 1.45–1.12 (m, 10H), 0.81 (t, *J* = 6.8 Hz, 3H). ^13^C NMR (101 MHz, CDCl_3_) δ: 142.4, 128.6, 128.5, 125.9, 71.6, 39.2, 37.7, 32.2, 32.0, 29.5, 25.7, 22.8, 14.2. *m*/*z*: 202 (M^+^-H_2_O, 32), 131 (50), 117 (47), 115 (18), 105 (22), 104 (92), 92 (23), 91 (100), 69 (17), 55 (13). HRMS (+ESI): *m*/*z* calculated for C_15_H_23_O [M-H]^+^: 219.1754, found: 219.1755. *ee* determination by chiral HPLC analysis, Phenomenex^®^ LUX Cellulose-1, Hex/*i*-PrOH 98:2, flow = 1 mL/min, T = RT, retention times: t_r_(*R*) = 16.1 min (major enantiomer), t_r_(*S*) = 27.6 min.

(*E,R*)-1-phenylnon-1-en-3-ol **(3ga):** [84] Obtained as a yellow oil after purification by column chromatography (Et_2_O/cyclohexane 2:8). 35% yield, 78% *ee*. [α]_D_^26^ = −66.7 (*c* 1.2, CH_2_Cl_2_). {^Lit^ [α]_D_^21^ = −5.6 (*c* 1.07, CHCl_3_) for 91% *ee*}. ^1^H NMR (400 MHz, CDCl_3_) δ: 7.40–7.36 (m, 2H), 7.33–7.28 (m, 2H), 7.24–7.20 (m, 1H), 6.56 (d, *J* = 15.8 Hz, 1H), 6.21 (dd, *J* = 15.9, 6.8 Hz, 1H), 4.35–4.18 (m, 1H), 1.76–1.51 (m, 3H), 1.42–1.17 (m, 7H), 0.87 (t, *J* = 6.8 Hz, 3H). ^13^C NMR (101 MHz, CDCl_3_) δ: 136.9, 132.7, 130.4, 128.7, 127.8, 126.6, 73.3, 37.5, 31.9, 29.4, 25.6, 22.8, 14.2. *m*/*z*: 218 (M^+^, 3), 148 (14), 134 (11), 133 (100), 131 (17), 130 (29), 129 (16), 128 (16), 115 (64), 113 (14), 105 (47), 104 (21), 103 (31), 91 (45) 79 (17), 78 (18), 77 (46), 55 (55), 51 (14). HRMS (ASAP) *m*/*z* calculated for C_15_H_23_O [M+H]^+^: 219.1749, found: 219.1749. *ee* determination by chiral HPLC analysis, Phenomenex^®^ LUX Cellulose-1, Hex/*i*-PrOH 98:2, flow = 1 mL/min, T = RT, retention times: t_r_(*R*) = 20.4 min (major enantiomer), t_r_(*S*) = 37.8 min.

(*R*)-1-phenylnon-1-yn-3-ol **(3ha):** [85] Obtained as a yellow oil after purification by column chromatography (Et_2_O/cyclohexane 2:8). 43ae’0% yield, 56% *ee*. [α]_D_^23^ = −22.2 (*c* 3.6, CH_2_Cl_2_). {^Lit^ [α]_D_^23^ = −1.5 (*c* 0.69, CHCl_3_) for 92% ee). ^1^H NMR (400 MHz, CDCl_3_) δ: 7.46–7.40 (m, 2H), 7.35–7.28 (m, 3H), 4.60 (t, *J* = 6.6 Hz, 1H), 2.18 (br s, 1H), 1.85–1.73 (m, 2H), 1.56–1.43 (m, 2H), 1.40–1.25 (m, 6H), 0.90 (t, *J* = 6.9 Hz, 3H). ^13^C NMR (101 MHz, CDCl_3_) δ: 131.8, 128.4, 122.8, 90.4, 84.9, 63.1, 38.0, 31.9, 29.1, 25.3, 22.7, 14.2. *m*/*z*: 216 (M^+^, 4), 198 (58), 155 (40), 154 (14), 152 (10), 142 (15), 141 (67), 139 (12), 129 (69), 128 (100), 127 (11), 115 (65), 105 (15), 103 (21), 102 (86), 91 (16), 77 (20), 76 (18), 75 (10), 74 (10), 70 (14), 55 (12). HRMS (+ESI): *m*/*z* calculated for C_15_H_19_O [M-H]^+^: 215.1436, found: 215.1445. *ee* determination by chiral HPLC analysis, Phenomenex^®^ LUX Cellulose-1, Hex/*i*-PrOH 97:3, flow = 1 mL/min, retention times: t_r_(*R*) = 15.0 min, (major enantiomer). t_r_(*S*) = 44.5 min.

(*R*)-1-cyclohexyl-6-phenylhexan-1-ol **(3ab):** Obtained as a yellow oil after purification by column chromatography (Et_2_O/cyclohexane 2:8). 33% yield, 68% *ee*. [α]_D_^23^ = +40 (c 0.4, CH_2_Cl_2_). FTIR (neat) V_max_: 3368, 3026, 2922, 2852, 1603, 1496, 1450, 1077, 977, 745, 697 cm^−1^. ^1^H NMR (400 MHz, CDCl_3_) δ: 7.23–7.17 (m, 2H), 7.15–7.08 (m, 3H), 3.36–3.19 (m, 1H), 2.55 (t, *J* = 7.7 Hz, 2H), 1.77–1.63 (m, 3H), 1.64–1.51 (m, 4H), 1.49–0.86 (m, 11H). ^13^C NMR (101 MHz, CDCl_3_) δ: 142.8, 128.5, 128.4, 125.7, 76.2, 43.7, 36.1, 34.0, 31.7, 29.3, 27.8, 26.6, 26.4, 26.3, 25.8. *m*/*z*: 228 (M^+^, 21) 145 (27), 128 (16), 132 (23), 117 (29), 105 (16), 104 (100), 95 (29), 92 (19), 91 (89) 83 (10), 81 (14), 67 (17), 55 (23). HRMS (+ESI): *m*/*z* calculated for C_17_H_26_O [M+Na]^+^: 269.1876, found: 269.1883. *ee* determination by chiral HPLC analysis, Phenomenex^®^ LUX Cellulose-1, Hex/*i*-PrOH 97:3 flow = 1 mL/min, T = RT, t_r_(*S*) = 9.67 min, t_r_(*R*) = 10.12 min (major enantiomer).

(*R*)-5-(*tert*-butyl-dimethyl-silanyloxy)-1-cyclohexylheptan-1-ol **(3ac):** Obtained as a brown oil after purification by column chromatography (Et_2_O/cyclohexane 3:7). 27% yield, 58% *ee*. [α]_D_^23^ = +10.81 (*c* 3.7, CH_2_Cl_2_). FTIR (neat) V_max_: 3369, 2927, 2854, 1428, 1106, 823, 699, 613, 503, 187 cm^−1^. ^1^H NMR (400 MHz, CDCl_3_) δ 7.67 (dd, *J* = 4.2, 3.4 Hz, 4H), 7.44–7.34 (m, 6H), 3.67 (t, *J* = 6.1 Hz, 2H), 3.36–3.29 (m, 1H), 1.83–1.70 (m, 3H), 1.70–0.88 (m with s at 1.05, 25H). ^13^C NMR (101 MHz, CDCl_3_) δ: 135.7, 134.2, 129.6, 127.7, 76.2, 63.9, 43.6, 33.9, 32.7, 29.4, 27.8, 27.0, 26.7, 26.5, 26.3, 22.2, 19.3. HRMS (ASAP): *m*/*z* calculated for C_27_H_41_O_2_Si [M+H]^+^: 425.2876, found: 425.2876. *ee* determination by chiral HPLC analysis, Phenomenex^®^ LUX Cellulose-1, Hex/*i*-PrOH 97:3, flow = 1 mL/min, T= RT retention times: t_r_(*R*) = 5.92 min (major enantiomer), t_r_(*S*) = 7.41 min.

(*R*)-5-bromo-1-cyclohexylpentan-1-ol **(3ad):** Obtained as a yellow oil after purification by column chromatography (Et_2_O/cyclohexane 1:1). 51% yield, 84% *ee* (determined on the corresponding benzoate **3ad’**). [α]_D_^25^ = +12 (*c* 1, CH_2_Cl_2_). FTIR (neat) V_max_: 3368, 2928, 2851, 1450, 1237, 1087, 1064, 1047, 975, 893, 562 cm^−1^. ^1^H NMR (400 MHz, CDCl_3_) δ: 3.41 (t, *J* = 6.9 Hz, 2H), 3.37–3.32 (m, 1H), 1.94–1.83 (m, 2H), 1.83–1.69 (m, 3H), 1.69–1.57 (m, 3H), 1.57–0.89 (m, 10H). ^13^C NMR (101 MHz, CDCl_3_) δ: 76.1, 43.8, 34.0, 33.3, 33.0, 29.4, 27.8, 26.6, 26.5, 26.3, 24.8. *m*/*z*: 230 (M^+^-H_2_O, 1), 167 (41), 165 (40), 113 (62), 96 (12), 95 (100), 85 (85), 84 (20), 83 (19), 82 (13), 68 (10), 67 (48), 57 (25), 55 (51). HRMS (+ESI): *m*/*z* calculated for C_11_H_21_O [M+Na]^+^: 271.0673, found: 271.0668.

(*R*)-5-chloro-1-cyclohexylpentan-1-ol **(3ae):** Obtained as a yellow oil after purification by column chromatography (Et_2_O/cyclohexane 1:1). 36% yield, 60% *ee* (determined on the corresponding benzoate **3ae’**). [α]_D_^25^ = +20 (*c* 0.8, CH_2_Cl_2_). FTIR (neat) V_max_: 3369, 2923, 2851, 1449, 1309, 1088, 1065, 977, 892, 734, 651 cm^−1^. ^1^H NMR (400 MHz, CDCl_3_) δ: 3.57–3.48 (m, 2H), 3.39–3.27 (m, 1H), 1.84–1.70 (m, 5H), 1.70–1.55 (m, 4H), 1.53–0.90 (m, 9H). ^13^C NMR (101 MHz, CDCl_3_) δ: 76.1, 45.2, 43.8, 33.4, 32.8, 29.4, 27.8, 26.6, 26.4, 26.3, 23.5. *m*/*z*: 186 (M^+^-H_2_O, 2), 123 (21), 121 (62), 120 (13), 113 (44), 101 (13), 96 (13), 95 (100), 85 (62), 84 (17), 82 (14), 81 (15), 67 (47), 57 (22), 55 (49). HRMS (+ESI): *m*/*z* calculated for C_11_H_20_OCl [M-H]^+^: 203.1197, found: 203.1206.

General procedure for the synthesis of benzoate derivatives **3ea’**, **3ad’** and **3ae’:** [67] The corresponding chiral aliphatic alcohol (**3ea**, **3ad** or **3ae**, 0.10 mmol) was dissolved in anhydrous DCM (1 mL, 0.1 M). Sequentially, at 0 °C, Et_3_N (28 μL, 0.2 mmol, 2.0 eq), benzoyl chloride (12 μL, 0.1 mmol) and DMAP (1.3 mg, 0.20 mmol, 2.0 eq) were added. The reaction mixture was stirred overnight at RT. The reaction was quenched with water (1 mL), extracted with Et_2_O (3 × 5 mL). The combined organic layers were dried over MgSO_4_ and concentrated under vacuum. The crude material was purified by flash silica gel chromatography.

(*R*)-tetradecan-7-yl benzoate **(3ea’):** Obtained as a white solid after purification by column chromatography (Et_2_O/cyclohexane 2:8). 55% yield, 74% *ee*. [α]_D_^23^ = +20 (*c* 1.2, CH_2_Cl_2_). M_p_ = 42–45 °C. FTIR (neat) V_max_: 3349, 2926, 2855, 190, 1723, 1211, 1936, 1014, 703 cm^−1^. ^1^H NMR (400 MHz, CDCl_3_) δ: 8.17 (d, *J* = 8.1 Hz, 2H), 7.76–7.57 (m, 1H), 7.53 (t, *J* = 7.7 Hz, 2H), 3.63–3.53 (m, 1H), 1.50–1.36 (m, 6H), 1.28 (br s, 16H), 0.88 (t, *J* = 6.3 Hz, 6H). ^13^C NMR (101 MHz, CDCl_3_) δ: 162.5, 134.7, 130.7, 129.0, 72.2, 37.6, 32.0, 29.8, 29.5, 29.5, 25.8, 25.8, 22.8, 22.8, 14.3. *m*/z: 281 (14), 208 (15), 207 (100), 105 (68), 77 (11). HRMS (+ESI): *m*/*z* calculated for C_21_H_35_O_2_ [M+H]^+^: 319.2637, found: 319.2630. *ee* determination by chiral HPLC analysis, Phenomenex^®^ LUX Cellulose-1, Hexane 100, flow = 1 mL/min, T = RT. retention times: t_r_(*S*) = 9.97 min, t_r_(*R*) = 10.27 min (major enantiomer).

(*R*)-5-bromo-1-cyclohexylpentyl benzoate **(3ad’):** Obtained as a yellow oil after purification by column chromatography (Et_2_O/cyclohexane 3:7). 19% yield, 84% *ee*. FTIR (neat) V_max_: 2927, 2854, 1716, 1450, 1273, 1113, 712 cm^−1^. [α]_D_^23^ = +24 (c 0.5, CH_2_Cl_2_). ^1^H NMR (400 MHz, CDCl_3_) δ: 8.09–8.01 (m, 2H), 7.58–7.53 (m, 1H), 7.45 (t, *J* = 7.6 Hz, 2H), 5.08–4.97 (m, 1H), 3.38 (t, *J* = 6.8 Hz, 1H), 1.95–1.00 (m, 5H). ^13^C NMR (101 MHz, CDCl_3_) δ: 166.5, 133.0, 130.7, 129.7, 128.5, 78.3, 45.0, 41.5, 32.6, 30.7, 29.3, 28.3, 26.5, 26.3, 26.2, 23.0. *m*/*z*: 313 (1), 232 (12), 230 (13), 122 (17), 109 (18), 105 (100), 96 (19), 95 (24), 82 (11), 81 (29), 79 (13), 77 (37) 67 (26), 55 (14). HRMS (+ESI): *m*/*z* calculated for C_18_H_25_O_2_Br [M+Na]^+^: 375.0936, found: 375.0952. *ee* determination by chiral HPLC analysis, Phenomenex^®^ LUX Cellulose-1, Hex/*i*-PrOH 99:1, flow = 1 mL/min, T = RT, retention times: t_r_(*S*) = 16.34 min, t_r_(*R*) = 17.40 min (major enantiomer).

(*R*)-5-chloro-1-cyclohexylpentyl benzoate **(3ae’):** Obtained as a yellow oil after purification by column chromatography (Et_2_O/cyclohexane 3:7). 27% yield, 60% *ee*. [α]_D_^23^ = +10 (*c* 0.4, CH_2_Cl_2_). FTIR (neat) V_max_: 2929, 2854, 1717, 1450, 1279, 1113, 1027, 801, 712 cm^−1^. ^1^H NMR (400 MHz, CDCl_3_) δ: 8.09–8.00 (m, 2H), 7.59–7.52 (m, 1H), 7.47–7.41 (m, 2H), 5.07–4.93 (m, 1H), 3.50 (t, *J* = 6.7 Hz, 2H), 1.88–1.57 (m, 10H), 1.54–1.42 (m, 2H), 1.36–0.98 (m, 5H). ^13^C NMR (101 MHz, CDCl_3_) δ: 166.5, 133.0, 130.8, 129.7, 128.5, 78.3, 44.9, 41.5, 32.6, 30.7, 29.9, 29.3, 28.2, 26.5, 26.3, 23.0. *m*/*z*: 281 (0.4), 186 (17), 109 (10), 105 (100), 96 (16), 81 (15), 77 (24) 67 (15), 55 (10). HRMS (+ESI): *m*/*z* calculated for C_18_H_25_O_2_Cl [M+Na]^+^: 331.1441, found: 331.1425. *ee* determination by chiral HPLC analysis, Phenomenex^®^ LUX Cellulose-1, Hex/*i*-PrOH 97:3, flow = 1 mL/min, T = RT, retention times: t_r_(*S*) = 15.64 min, t_r_(*R*) = 16.12 min (major enantiomer).

## Figures and Tables

**Table 1:**
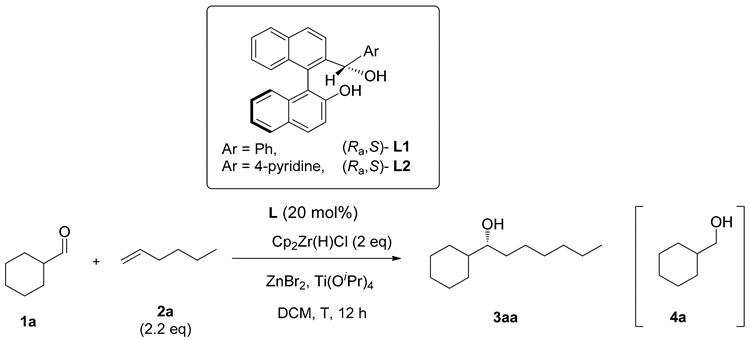
Optimisation for the synthesis of **3aa** ^a^.

Entry	L	T (°C)	Ti(O*^i^*Pr)_4_ (eq)	ZnBr_2_ (eq)	Conv. 3aa (%) ^b^	Conv. 4a (%) ^b^	ee (%) ^c^
1.	**L1**	35	1.5	0	0	>99	-
2.	**L1**	35	1.5	0.025	0	>99	-
3.	**L1**	35	1.5	0.5	99	1	86
4.	**L1**	35	1.5	1	0	>99	-
5.	**L1**	35	1.5	1.5	95	1	18
6.	**L1**	35	2	0.5	24	75	48
7.	**L1**	35	1	0.5	0	>99	-
8.	**L1**	35	2	1	97	1	58
9.	**L1**	35	2	2	94	2	52
10.	**L1**	r.t.	1.5	0.5	36	64	70
11.	**L1**	35	1.5	- ^d^	99	1	64
12.	**L1**	r.t.	1.5	- ^d^	85	15	40
13.	**L2**	35	1.5	0.5	32	62	8
14.	**L1**	35	-	0.5	0	11	-
15.		35	1.5	0.5	0	>99	-

^a^ Reaction conditions: **1a** (0.3 mmol), **2a** (2.2 eq., 0.66 mmol), Cp_2_Zr(H)Cl (2.0 eq, 0.60 mmol), **L1** or **L2** (20 mol %), Ti(O*^i^*Pr)_4_ (x eq), ZnBr_2_ (x eq), DCM (0.3 + 0.1 mL), T, 12 h. ^b^ Determined by GC-MS. ^c^ Determined by Chiral GC (see experimental section for further details). ^d^ Reaction carried out with CuCl (1.5 eq) instead of ZnBr_2_.

**Table 2:**
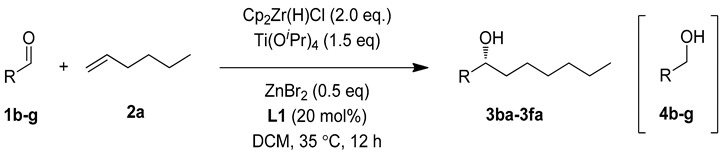
Catalytic enantioselective addition of 1-hexene (**2a**) to aliphatic aldehydes (**1b–g**) ^a^.

Entry	Product	Conv. to 3 (%) ^b^	Conv. to by-Product 4 (%) ^b^	Yield (%) ^c^	ee (%) ^d^
1.	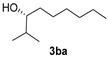	>99	0	42	76(*R*) ^e^
2.	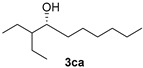	>99	0	54	70(*R*) ^e^
3.	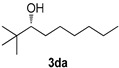	93	7	50	84(*R*) ^e^
4.	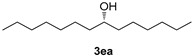	91	4	48	74(*R*) ^f,g^
5.	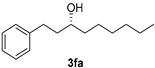	>99	0	40	74(*R*) ^f^
6.	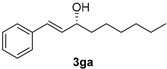	87	13	35	78(*R*) ^f^
7.	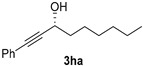	53	49	40	56(*R*) ^f^

^a^ Reaction conditions: **1b–f**, (0.3 mmol), **2a** (2.2 eq), **L1** (20 mol%), Cp_2_Zr(H)Cl (2.0 eq), Ti(O*^i^*Pr)_4_ (1.5 eq), ZnBr_2_ (0.5 eq), DCM (0.3 + 0.1 mL) 35 °C, 12 h. ^b^ Determined by GC-MS. ^c^ Isolated yield after flash chromatography. ^d^ Configuration in brackets assigned by comparison with optical rotation values in literature (see experimental section for further details). ^e^ Determined by Chiral GC (see experimental section for further details). ^f^ Determined by Chiral HPLC (see experimental section for further details). ^g^ Determined on the corresponding benzoate derivative (see experimental section for further details).

**Table 3:**
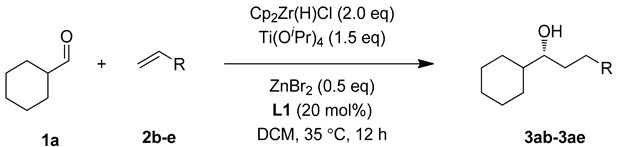
Catalytic enantioselective addition of alkenes (**2b–e**) to cyclohexanecarboxaldehyde (**1a**) ^a^.

Entry	Product	Conv. (%) ^b^	Conv. to Reduced Product 4a (%) ^b^	Yield (%) ^c^	ee (%) ^d^
1	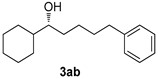	60	39	33	68(*R*) ^e^
2	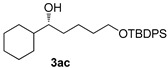	n.d	n.d.	27	58(*R*) ^e^
3	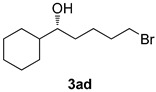	73	20	51	84(*R*) ^e,f^
4	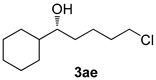	66	34	36	60(*R*) ^e,f^

^a^ Reaction conditions: **1a** (0.3 mmol), **2b–e** (2.2 eq), **L1** (20 mol %), Cp_2_Zr(H)Cl (2.0 eq), Ti(O*^i^*Pr)_4_ (1.5 eq), ZnBr_2_ (0.5 eq), DCM (0.3 + 0.1 mL) 35 °C, 12 h. ^b^ Determined by GC-MS. ^c^ Isolated yield after flash chromatography. ^d^ Configuration in brackets assigned by comparison of the optical rotation values in the literature (see experimental section for further details). ^e^ Determined by chiral HPLC (see experimental section for further details). ^f^ Determined on the corresponding benzoate derivative (see experimental section for further details).

## Data Availability

Not applicable.

## Supplementary Materials

The Supplementary Materials are available online. Spectroscopic data (IR, ^1^H and ^13^C NMR) for new compounds **3ca**, **3ea**, **3ab**, **3ac**, **3ad**, **3ae**, **3ea’**, **3ad’**, **3ae’**, and chiral GC and HPLC chromatograms for all compounds **3**.

## Sample Availability

Samples of the compounds **3** are available from the authors.
