# Supplementary material for: Catalytic Enantioselective Addition of Alkylzirconium Reagents to Aliphatic Aldehydes
Source: Molecules. 2021 Jul 24;26(15):4471. doi: 10.3390/molecules26154471 (PMC8347741; doi:10.3390/molecules26154471)

Supplementary Materials: Catalytic Enantioselective Addition of Alkylzirconium Reagents to Aliphatic Aldehydes

Jade Vaccari, María José González-Soria, Nicholas Carter and Beatriz Maciá *

Division of Chemistry & Environmental Science, Manchester Metropolitan University, Oxford Road, Manchester M1 5GD, UK; JADE.VACCARI@stu.mmu.ac.uk (J.V.); mariajo.gonzalez.s@gmail.com (M.J.G.-S.); ncarter1990@gmail.com (N.C.)
* Correspondence: b.macia-ruiz@mmu.ac.uk; Tel.: +44-(0)161-247-1416

Table of contents:

GC and HPLC data S-2

NMR spectra for new compounds S-14

IR spectra for new compounds S-23

GC and HPLC data

(*R*)-1-cyclohexylheptan-1-ol (3aa):

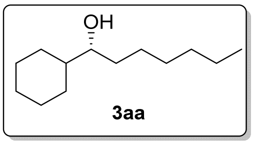

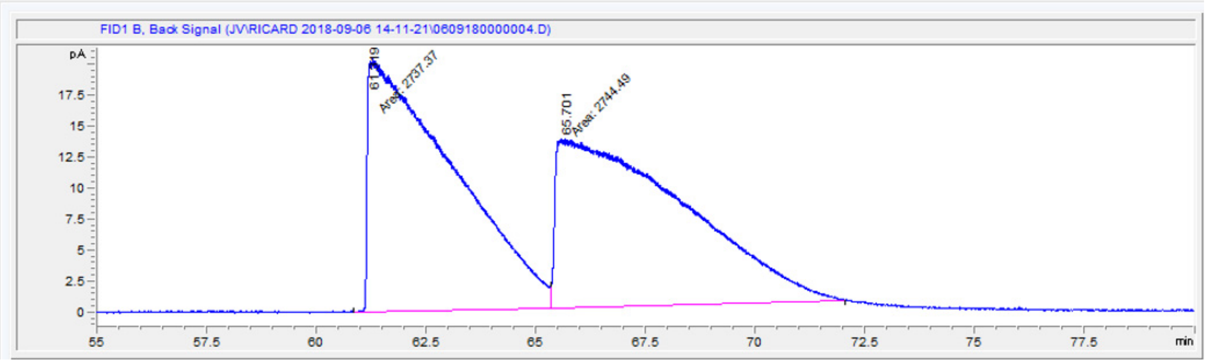

#	Time	Area	Height	Width	Area%	Symmetry
1	61.319	2737.4	20.4	2.2384	49.935	0
2	65.701	2744.5	13.6	3.367	50.065	8.35E-2

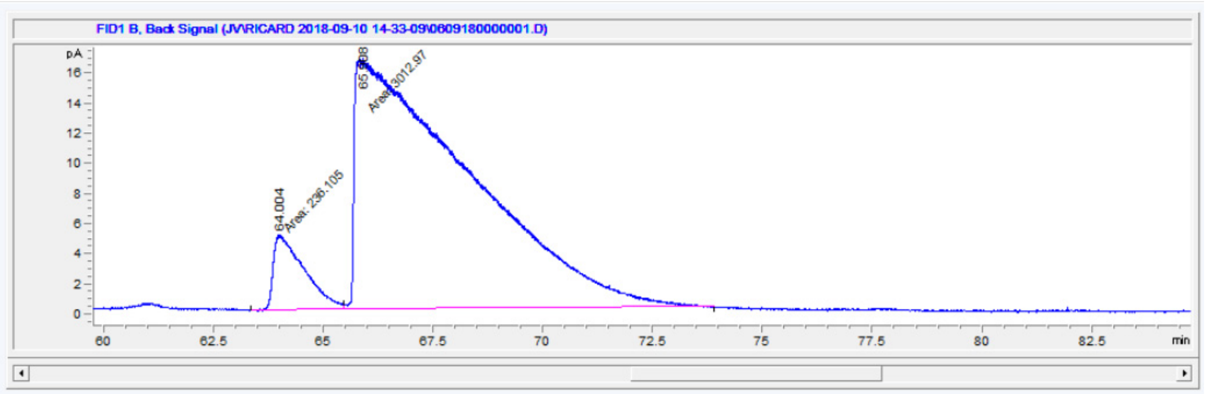

#	Time	Area	Height	Width	Area%	Symmetry
1	64.004	236.1	5	0.7948	7.267	0
2	65.908	3013	16.6	3.0199	92.733	8.07E-2

(*R*)-2-methylnonan-3-ol (3ba):

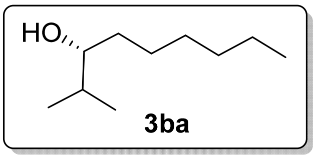

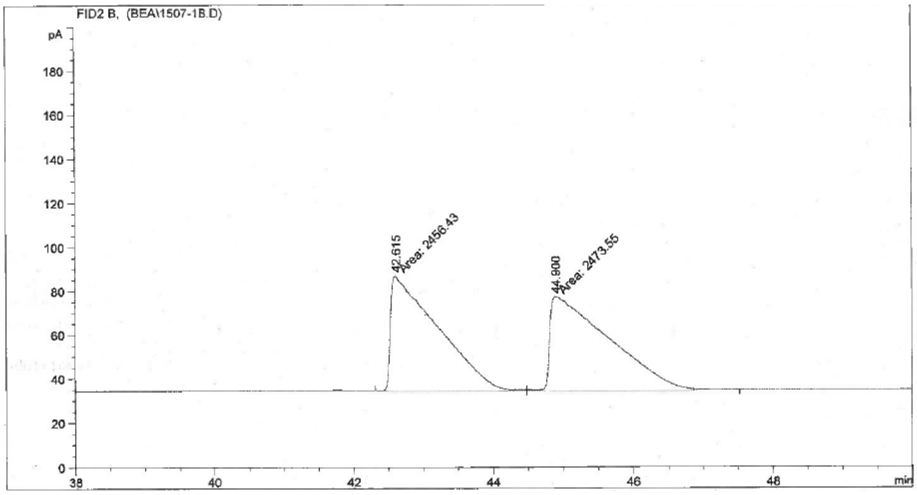

Peak #	RetTime [min]	Type	Width [min]	Area [pA*s]	Height [pA]	Area %
1	42.615	MF	0.7795	2456.43433	52.52231	49.82640
2	44.900	FM	0.9662	2473.55078	42.66872	50.17360

Totals : 4929.98511 95.19103

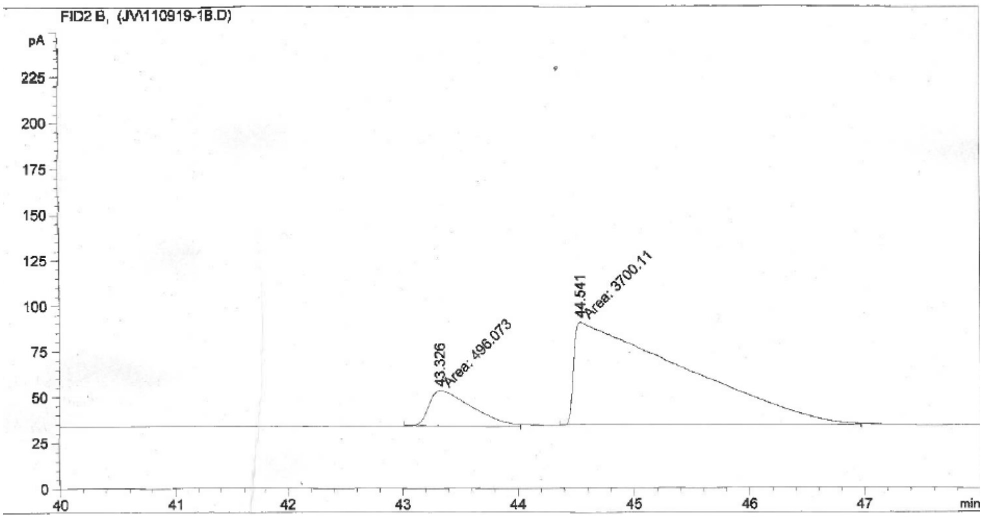

Peak #	RetTime [min]	Type	Width [min]	Area [pA*s]	Height [pA]	Area %
1	43.326	MM	0.4279	496.07318	19.32008	11.82200
2	44.541	MM	1.0994	3700.11230	56.09291	88.17800

Totals : 4196.18549 75.41299

(*R*)-3-ethylnonan-4-ol (3ca):

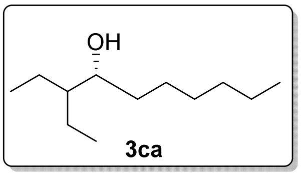

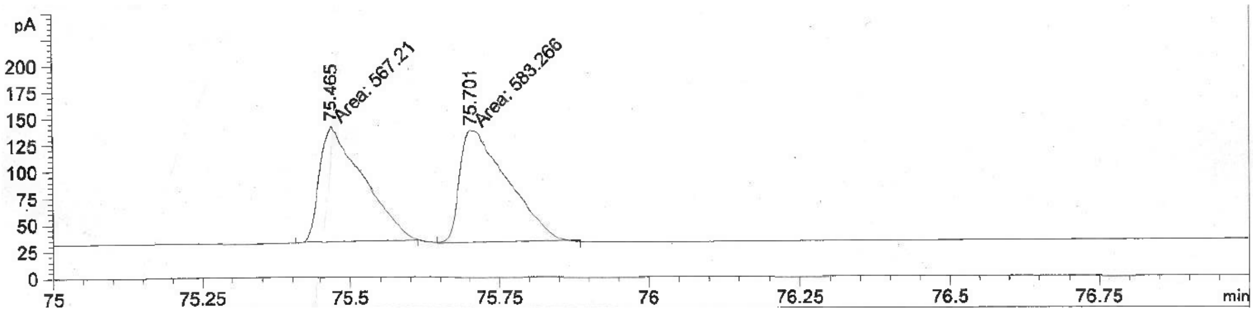

Peak #	RetTime [min]	Type	Width [min]	Area [pA*s]	Height [pA]	Area %
1	75.465	MM	0.0876	567.20984	107.85661	49.30220
2	75.701	MM	0.0930	583.26581	104.49026	50.69780
Totals :				1150.47565	212.34687	

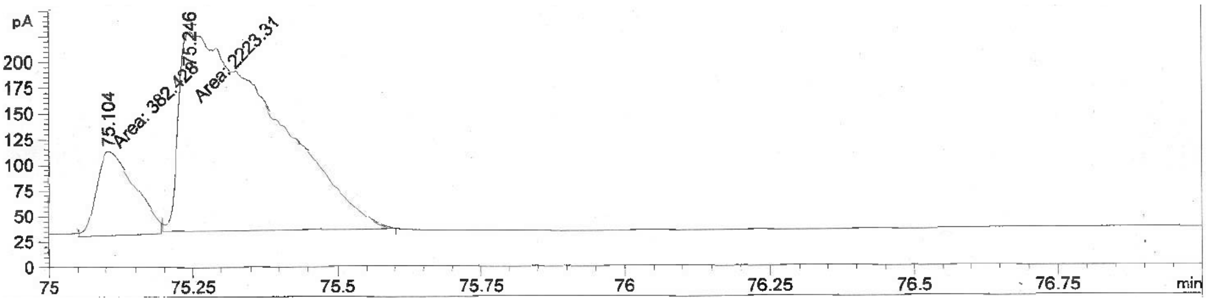

Peak #	RetTime [min]	Type	Width [min]	Area [pA*s]	Height [pA]	Area %
1	75.104	MM	0.0781	382.42804	81.57475	14.67638
2	75.246	MM	0.1916	2223.31079	193.39082	85.32362
Totals :				2605.73883	274.96558	

(*R*)-2,2-dimethylnonan-3-ol (3da):

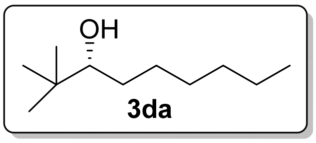

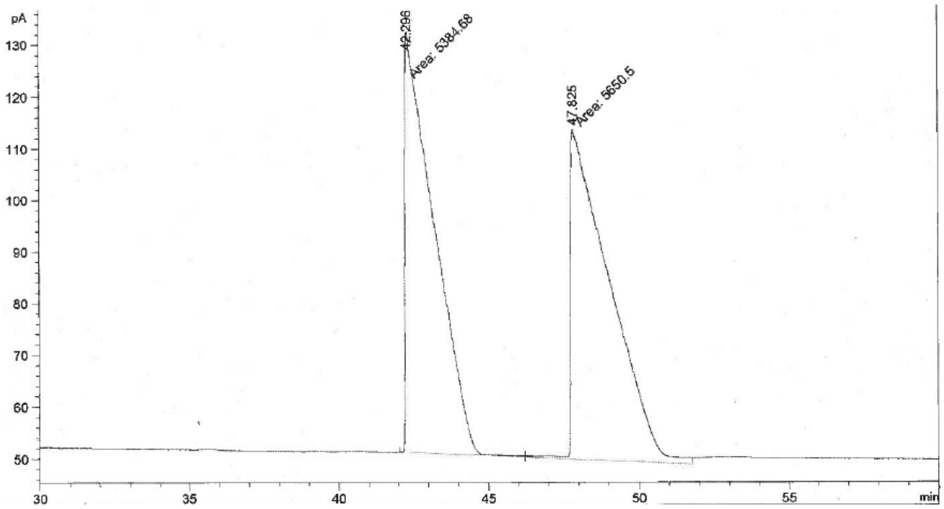

Peak #	RetTime [min]	Type	Width [min]	Area [pA*s]	Height [pA]	Area %
1	42.296	MF	1.1055	5384.67529	81.17917	48.79557
2	47.825	FM	1.4805	5650.49805	63.60886	51.20443

Totals : 1.10352e4 144.78802

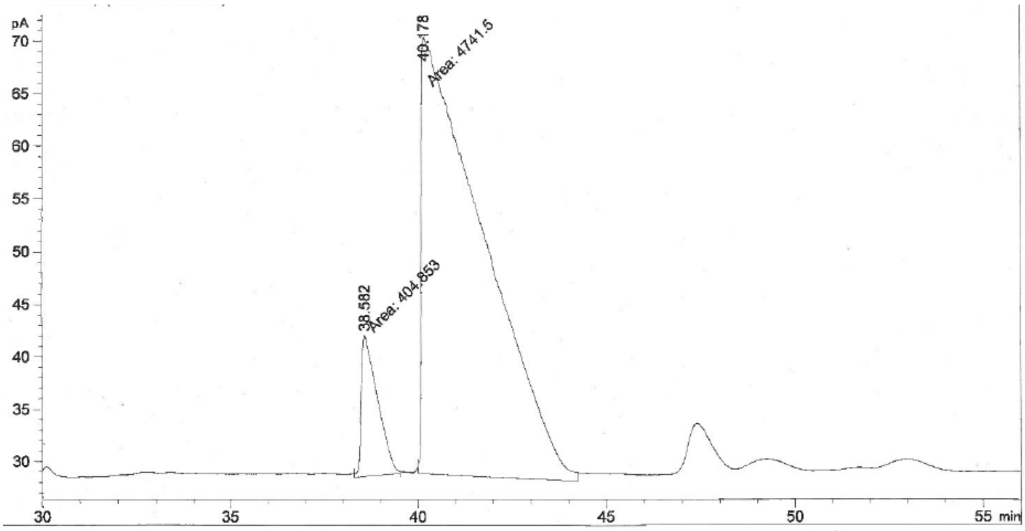

Peak #	RetTime [min]	Type	Width [min]	Area [pA*s]	Height [pA]	Area %
1	38.582	MM	0.5006	404.85330	13.48012	7.86680
2	40.178	MM	1.8991	4741.49707	41.61275	92.13320

Totals : 5146.35037 55.09288

(*R*)-1-phenylnonan-3-ol (3fa):

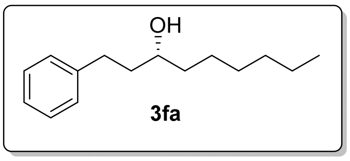

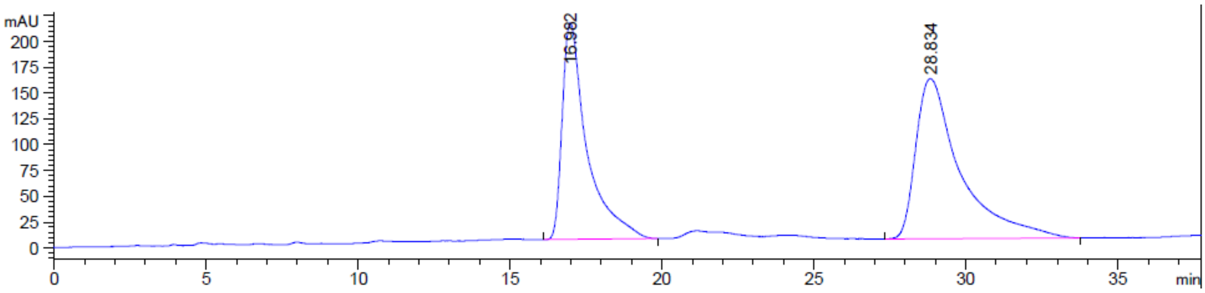

Peak #	RetTime [min]	Type	Width [min]	Area [mAU*s]	Height [mAU]	Area %
1	16.982	MM	0.9759	3.55032e4	606.35913	50.2469
2	28.831	MM	1.6395	3.51543e4	357.37430	49.7531
Totals :				7.06575e4	963.73343	

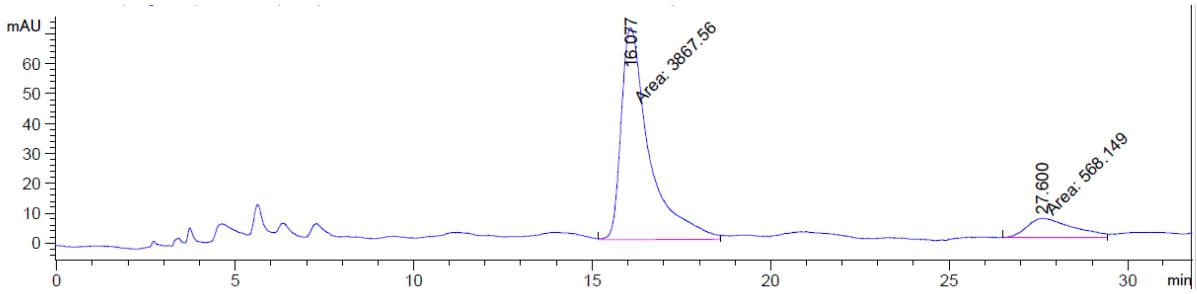

Peak #	RetTime [min]	Type	Width [min]	Area [mAU*s]	Height [mAU]	Area %
1	16.077	MM	0.9071	3867.55762	71.05953	87.1915
2	27.600	MM	1.4819	568.14905	6.38987	12.8085
Totals :				4435.70667	77.44940	

(*E*,*R*)-1-phenylnon-1-en-3-ol (3ga):

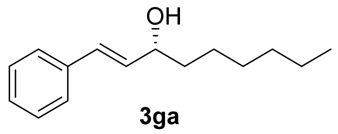

3ga

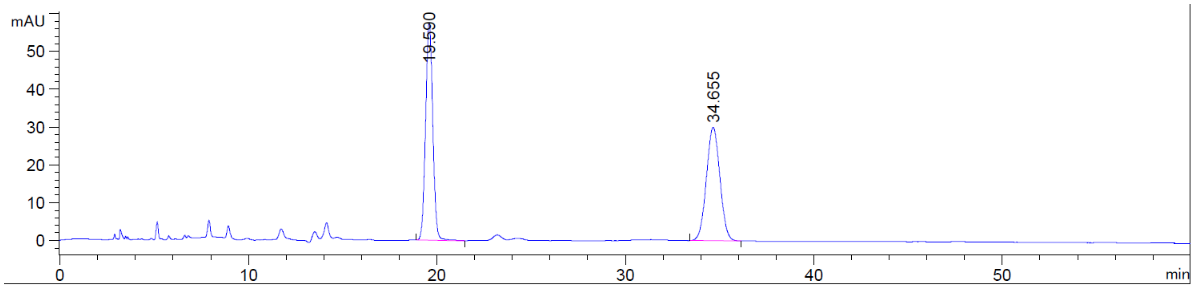

Signal 3: DAD1 C, Sig=210,8 Ref=360,100

Peak #	RetTime [min]	Type	Width [min]	Area [mAU*s]	Height [mAU]	Area %
1	19.590	BB	0.4084	3951.60498	150.48300	50.0061
2	34.657	BB	0.7707	3950.63721	78.82470	49.9939

Totals : 7902.24219 229.30770

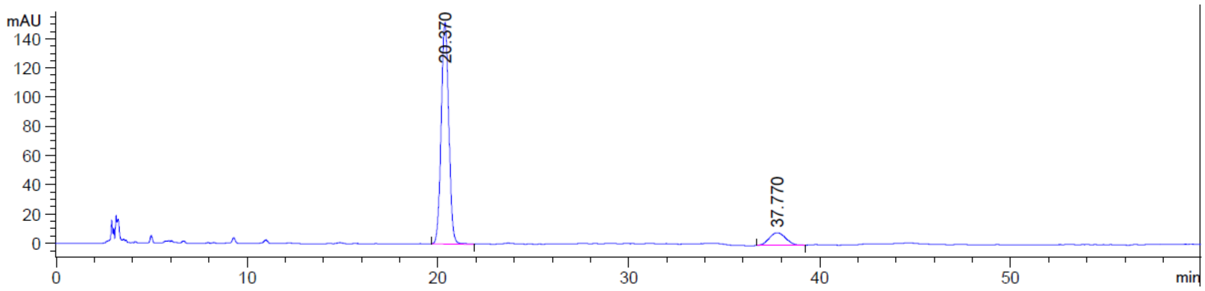

Peak #	RetTime [min]	Type	Width [min]	Area [mAU*s]	Height [mAU]	Area %
1	20.370	BB	0.4321	4175.67285	151.22928	88.8326
2	37.770	BB	0.7103	524.93573	8.69593	11.1674

Totals : 4700.60858 159.92521

(*R*)-1-phenylnon-1-yn-3-ol (3ha):

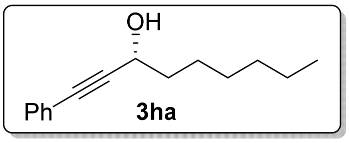

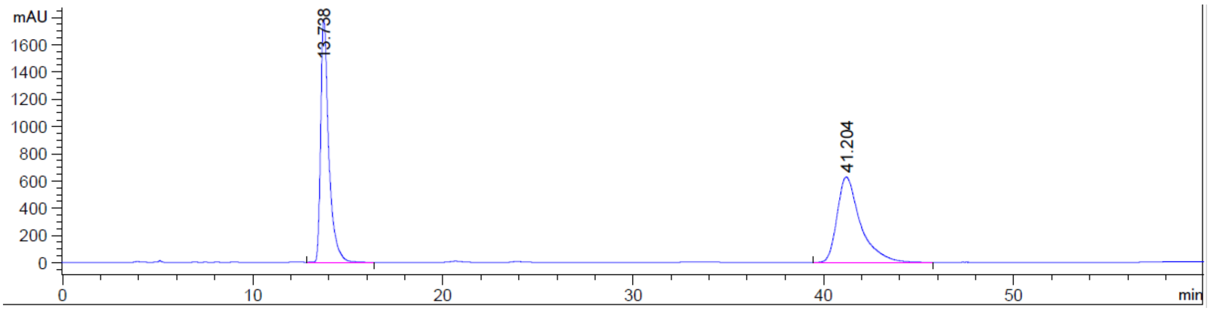

Peak #	RetTime [min]	Type	Width [min]	Area [mAU*s]	Height [mAU]	Area %
1	13.738	BB	0.4350	3.67948e4	1244.30371	50.2286
2	41.203	BB	1.0279	3.64599e4	427.96091	49.7714
Totals :				7.32547e4	1672.26462	

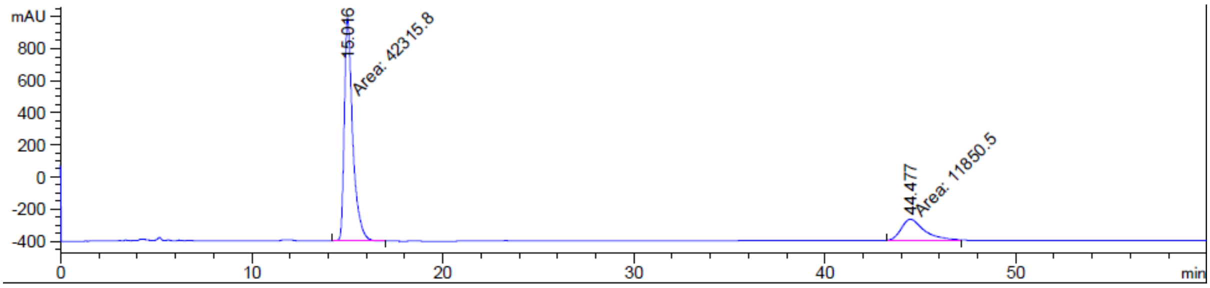

Peak #	RetTime [min]	Type	Width [min]	Area [mAU*s]	Height [mAU]	Area %
1	15.016	MM	0.5079	4.23158e4	1388.54138	78.1220
2	44.477	MM	1.4777	1.18505e4	133.65500	21.8780
Totals :				5.41663e4	1522.19638	

(*R*)-1-cyclohexyl-6-phenylhexan-1-ol (3ab):

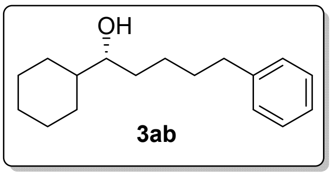

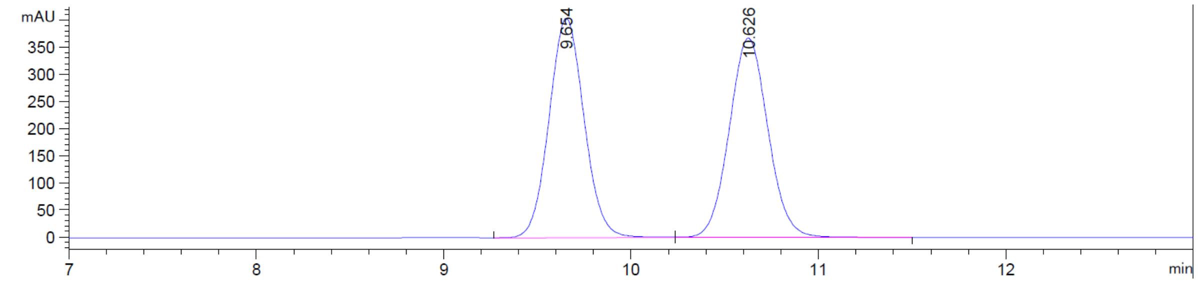

Peak #	RetTime [min]	Type	Width [min]	Area [mAU*s]	Height [mAU]	Area %
1	9.654	BV	0.2019	5277.85205	405.38049	49.8557
2	10.626	VB	0.2241	5308.40869	368.46069	50.1443

Totals : 1.05863e4 773.84119

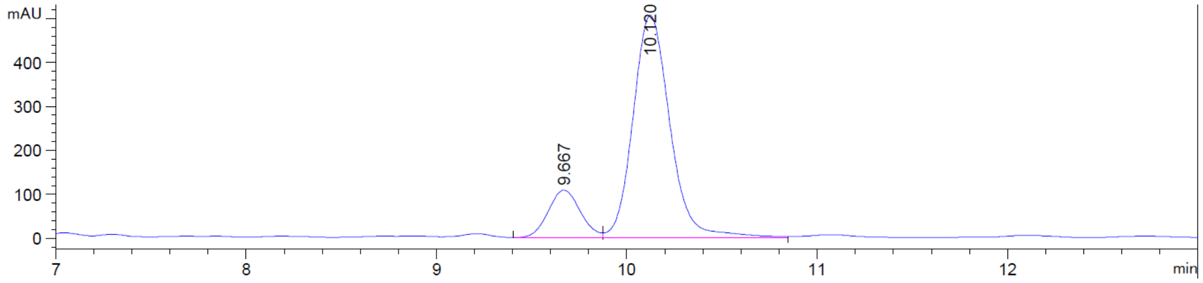

Peak #	RetTime [min]	Type	Width [min]	Area [mAU*s]	Height [mAU]	Area %
1	9.667	BV	0.1802	1261.65015	108.10708	15.6404
2	10.120	VV	0.2089	6804.94336	505.95508	84.3596

Totals : 8066.59351 614.06216

(*R*)-5-(*tert*-butyl-dimethyl-silanyloxy)-1-cyclohexylheptan-1-ol (3ac):

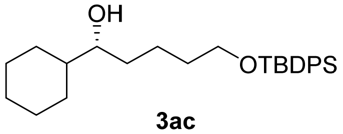

3ac

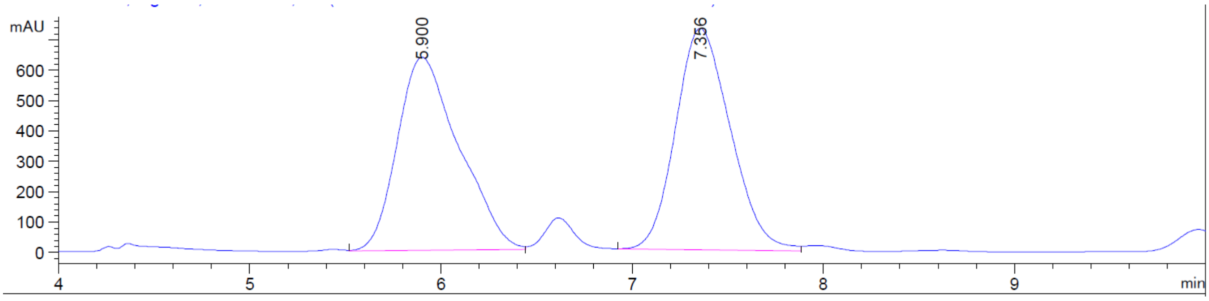

Peak #	RetTime [min]	Type	Width [min]	Area [mAU*s]	Height [mAU]	Area %
1	5.900	VV	0.3354	1.96872e4	865.40845	49.5349
2	7.355	VB	0.3121	2.00569e4	982.68854	50.4651
Totals :				3.97440e4	1848.09698	

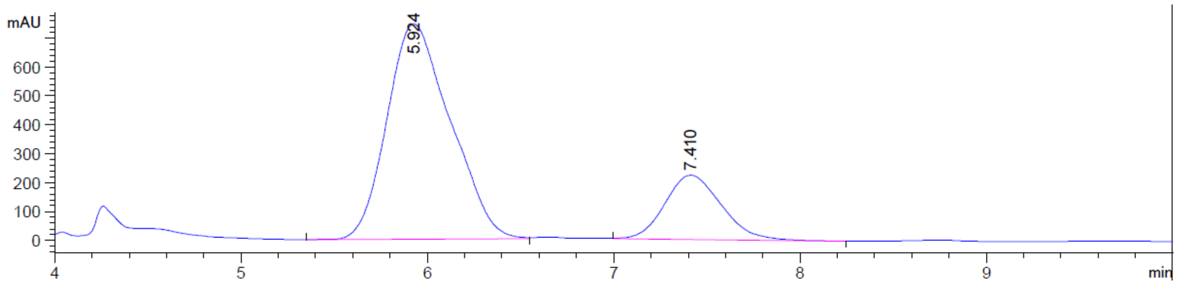

Peak #	RetTime [min]	Type	Width [min]	Area [mAU*s]	Height [mAU]	Area %
1	5.924	BV	0.3497	1.76293e4	745.98334	78.8851
2	7.410	VB	0.3285	4718.76367	223.34132	21.1149
Totals :				2.23481e4	969.32466	

(*R*)-tetradecan-7-yl benzoate (3ea′):

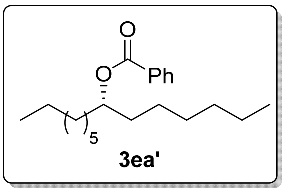

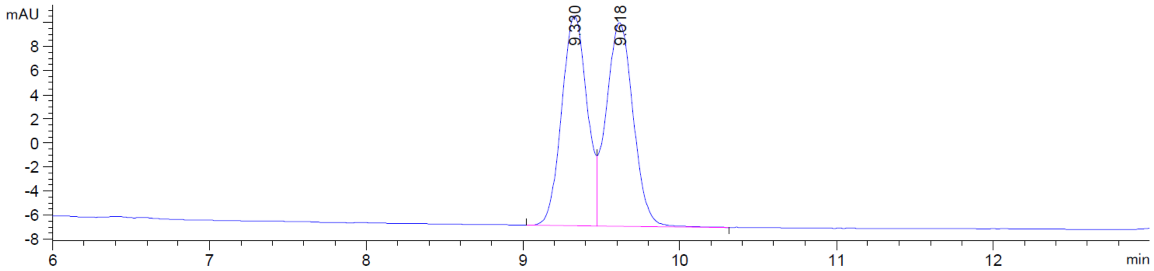

Peak #	RetTime [min]	Type	Width [min]	Area [mAU*s]	Height [mAU]	Area %
1	9.330	BV	0.1753	196.40424	17.44556	49.2373
2	9.618	VB	0.1856	202.48932	16.92315	50.7627
Totals :				398.89355	34.36871	

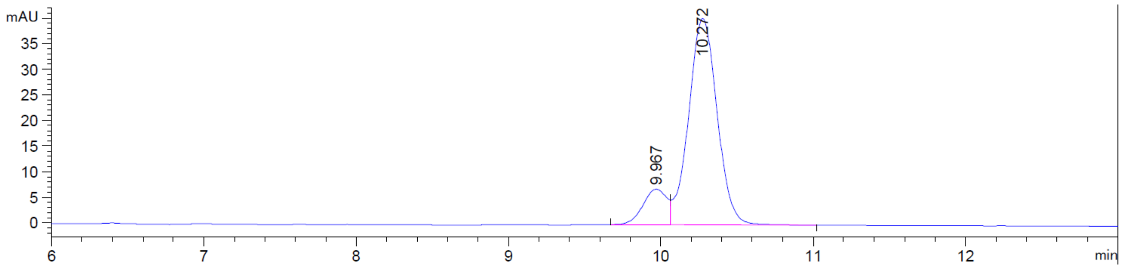

Peak #	RetTime [min]	Type	Width [min]	Area [mAU*s]	Height [mAU]	Area %
1	9.967	BV	0.1684	76.31265	6.93274	12.7117
2	10.272	VB	0.1994	524.01904	40.37003	87.2883
Totals :				600.33170	47.30276	

(*R*)-5-bromo-1-cyclohexylpentyl benzoate (3ad′):

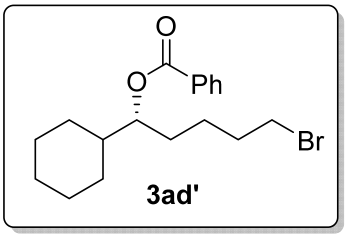

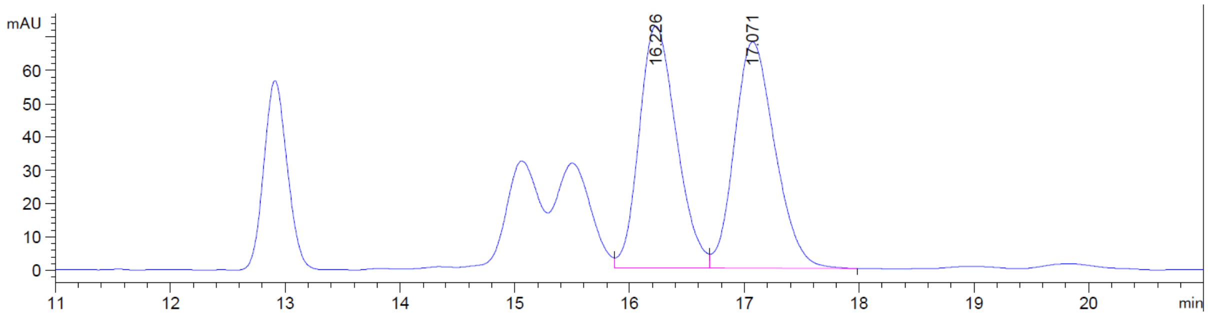

Peak #	RetTime [min]	Type	Width [min]	Area [mAU*s]	Height [mAU]	Area %
1	16.226	VV	0.3475	1638.97668	72.55424	49.6177
2	17.071	VB	0.3735	1664.23022	68.01123	50.3823
Totals :				3303.20691	140.56547	

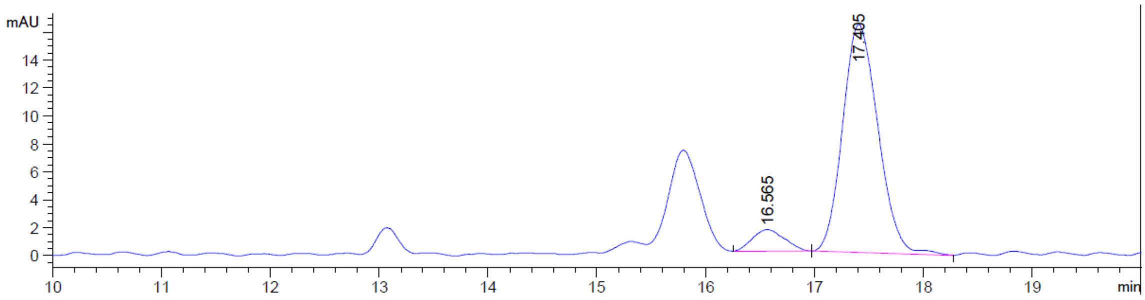

Peak #	RetTime [min]	Type	Width [min]	Area [mAU*s]	Height [mAU]	Area %
1	16.565	BB	0.3089	31.88211	1.54374	7.9445
2	17.405	BB	0.3581	369.42731	16.31731	92.0555
Totals :				401.30942	17.86105	

(*R*)-5-chloro-1-cyclohexylpentyl benzoate (3ae′):

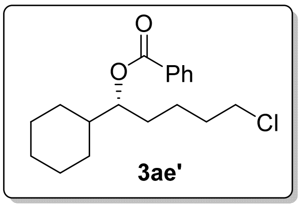

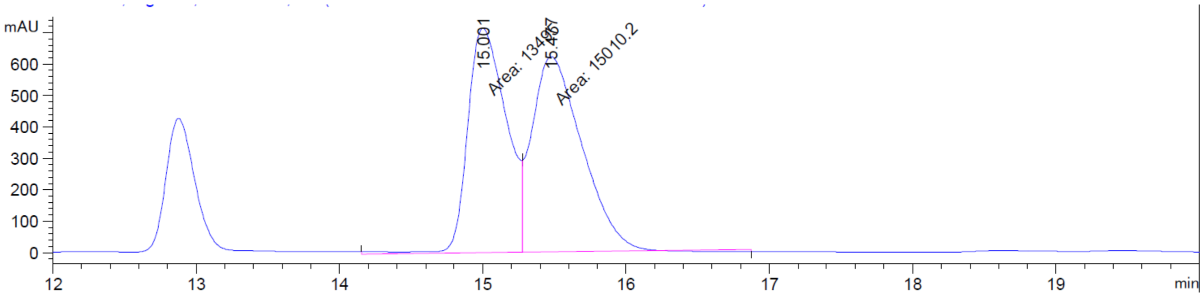

Peak #	RetTime [min]	Type	Width [min]	Area [mAU*s]	Height [mAU]	Area %
1	14.375	BV	0.2793	2733.23926	149.50731	46.2404
2	14.760	VB	0.3447	3177.69678	138.99434	53.7596
Totals :				5910.93604	288.50165	

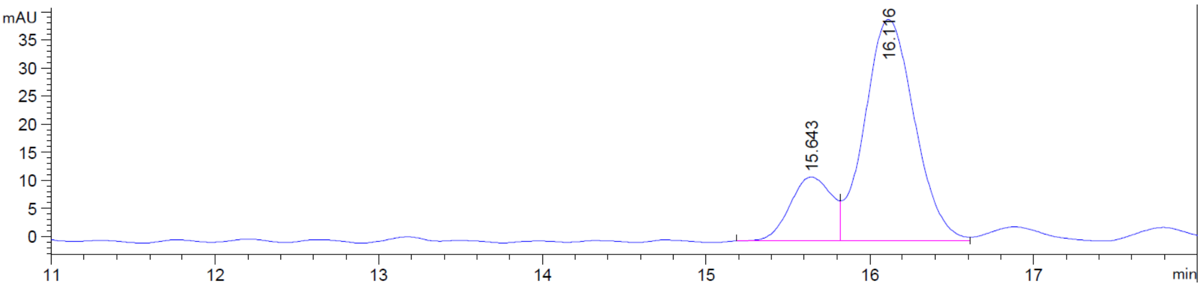

Peak #	RetTime [min]	Type	Width [min]	Area [mAU*s]	Height [mAU]	Area %
1	15.643	BV	0.2737	203.69231	11.44197	19.5744
2	16.116	VV	0.3272	836.91541	39.50351	80.4256
Totals :				1040.60771	50.94548	

NMR spectra for new compounds

(*R*)-3-ethylnonan-4-ol (3ca):

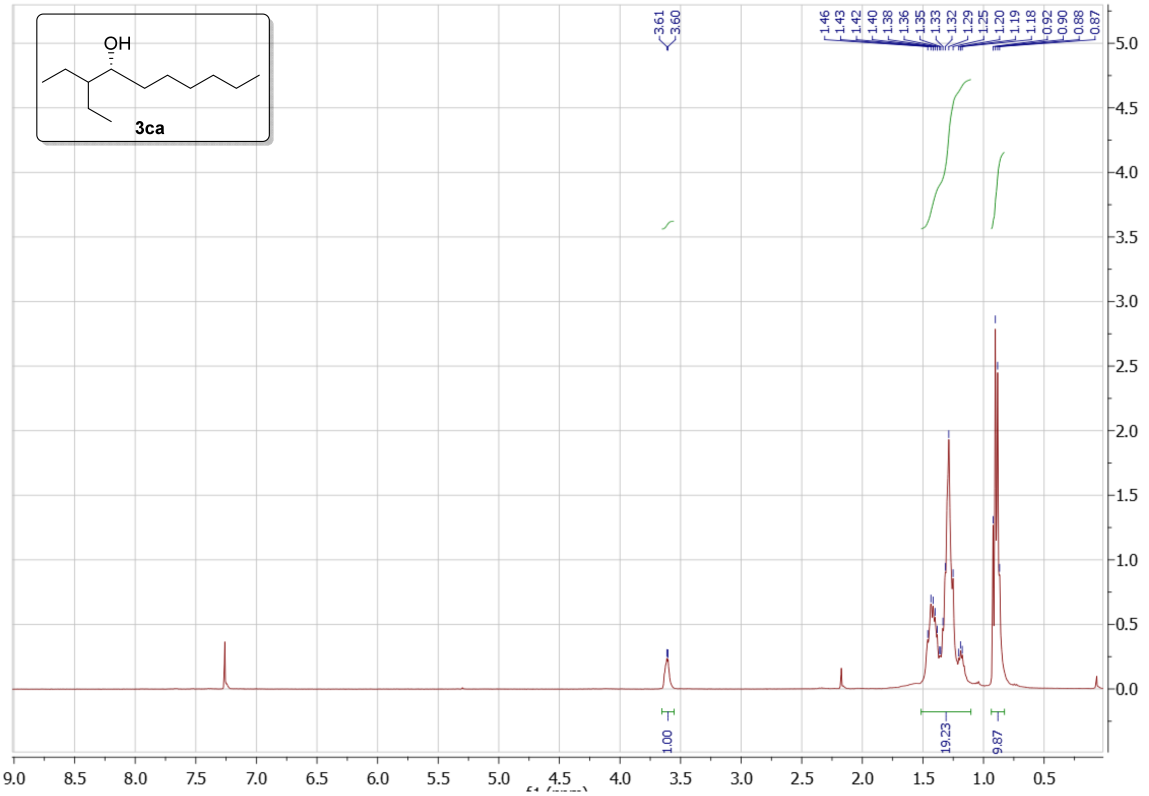

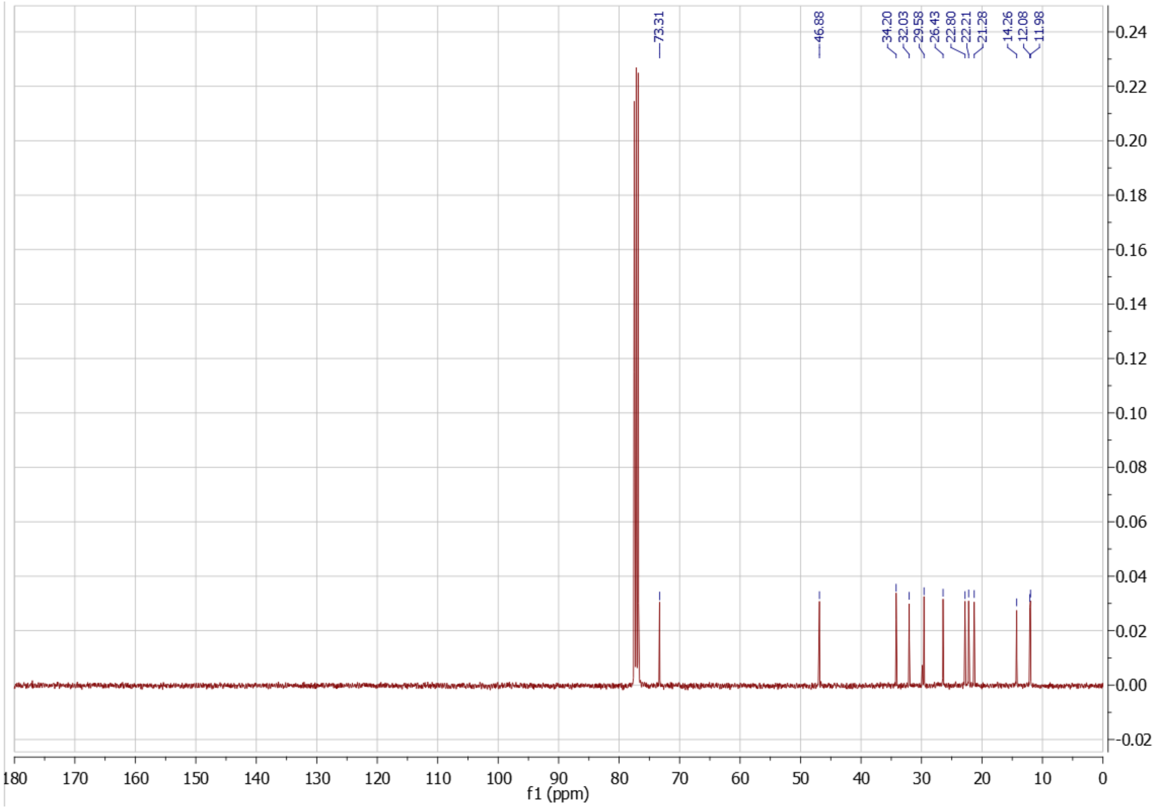

(*R*)-tetradecan-7-ol (3ea):

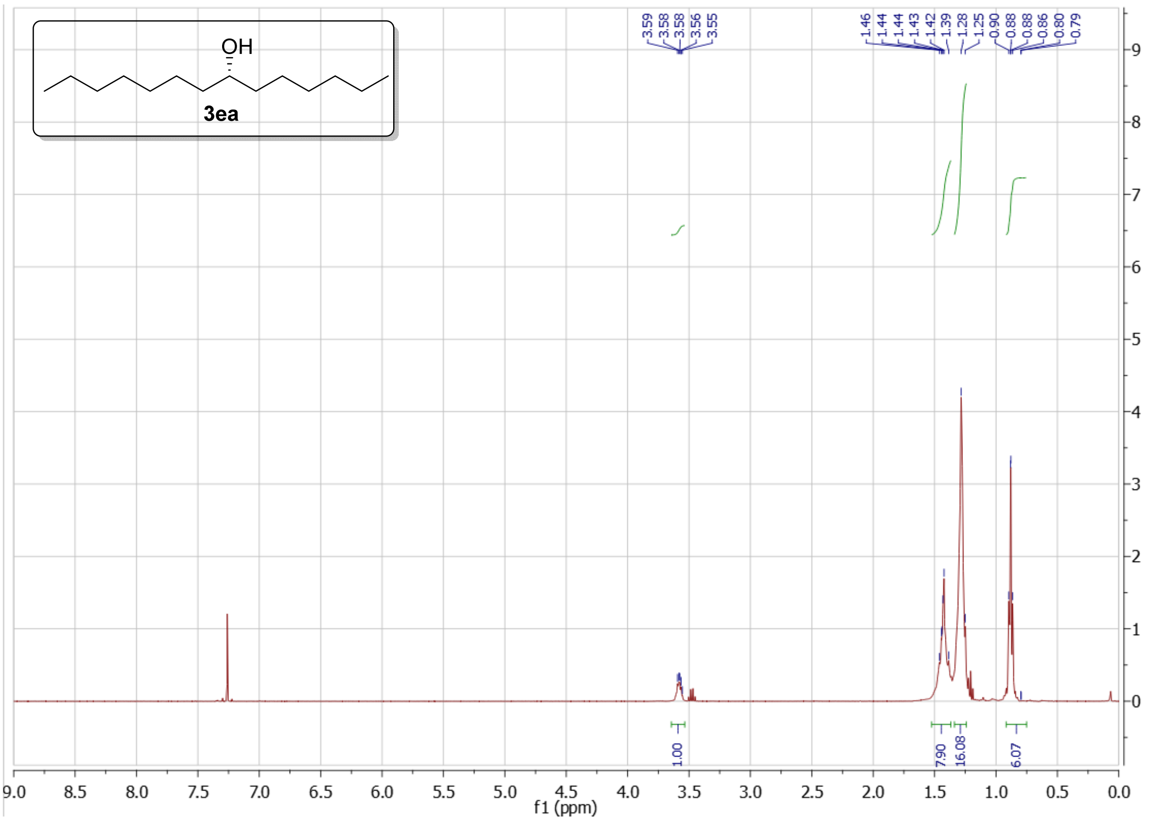

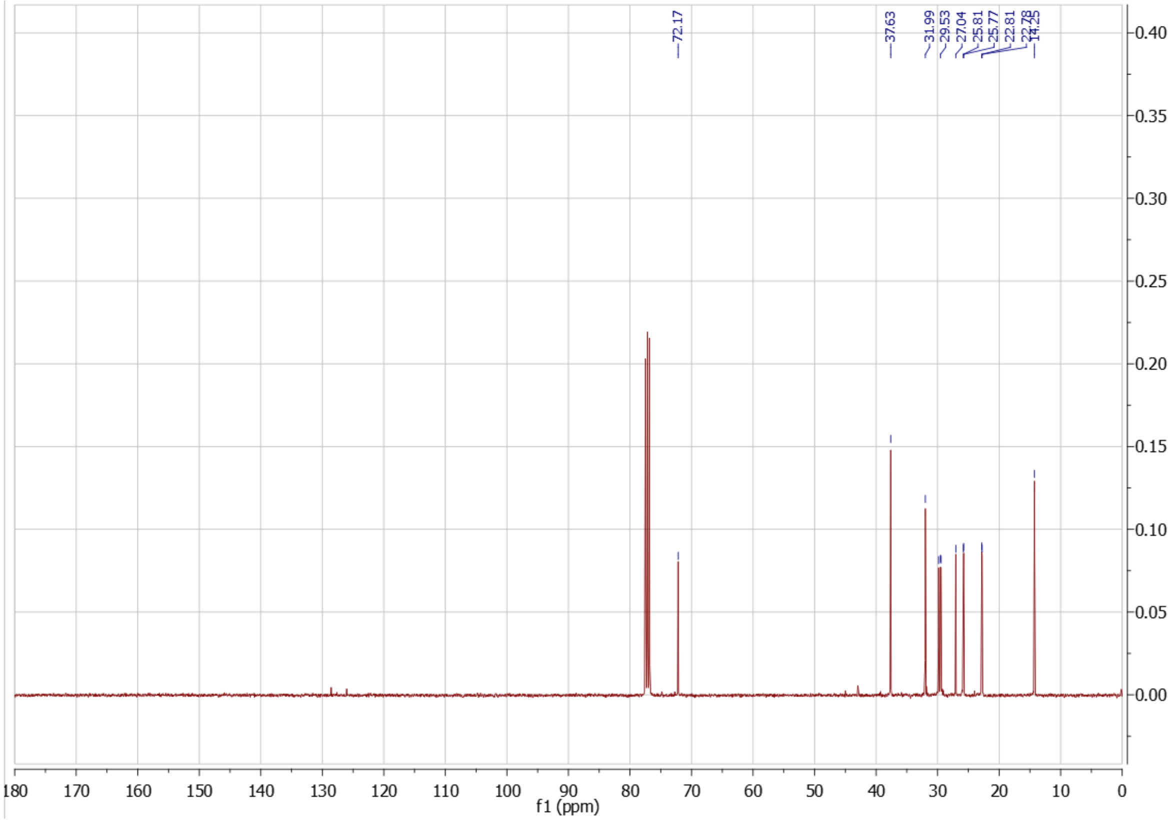

(*R*)-1-cyclohexyl-6-phenylhexan-1-ol (3ab):

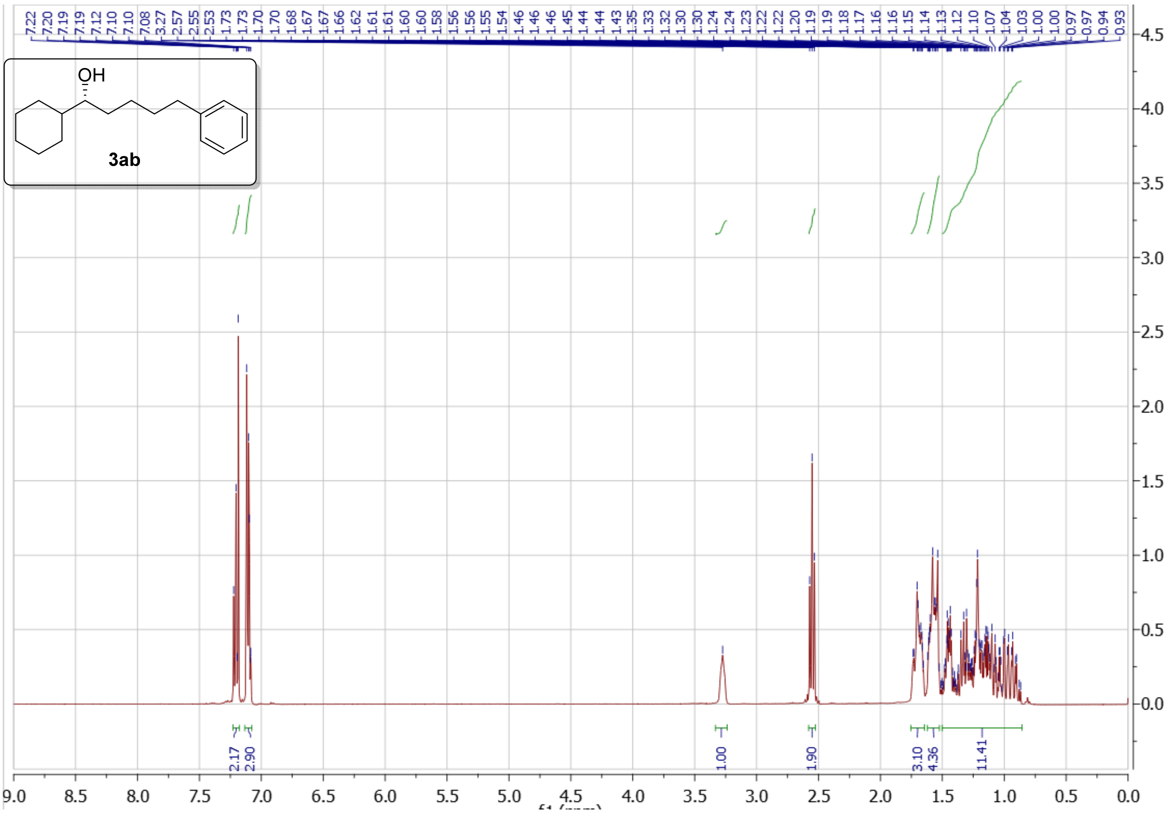

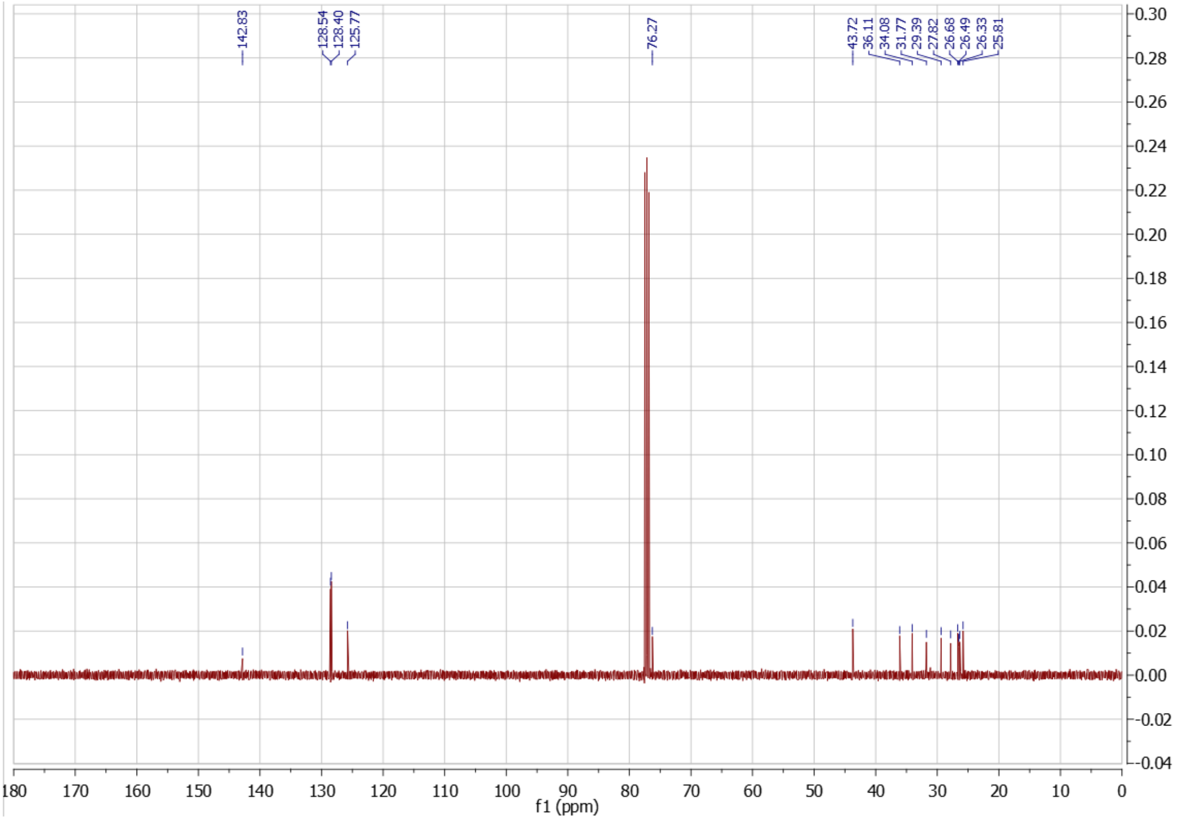

(*R*)-5-(*tert*-butyl-dimethyl-silanyloxy)-1-cyclohexylheptan-1-ol (3ac):

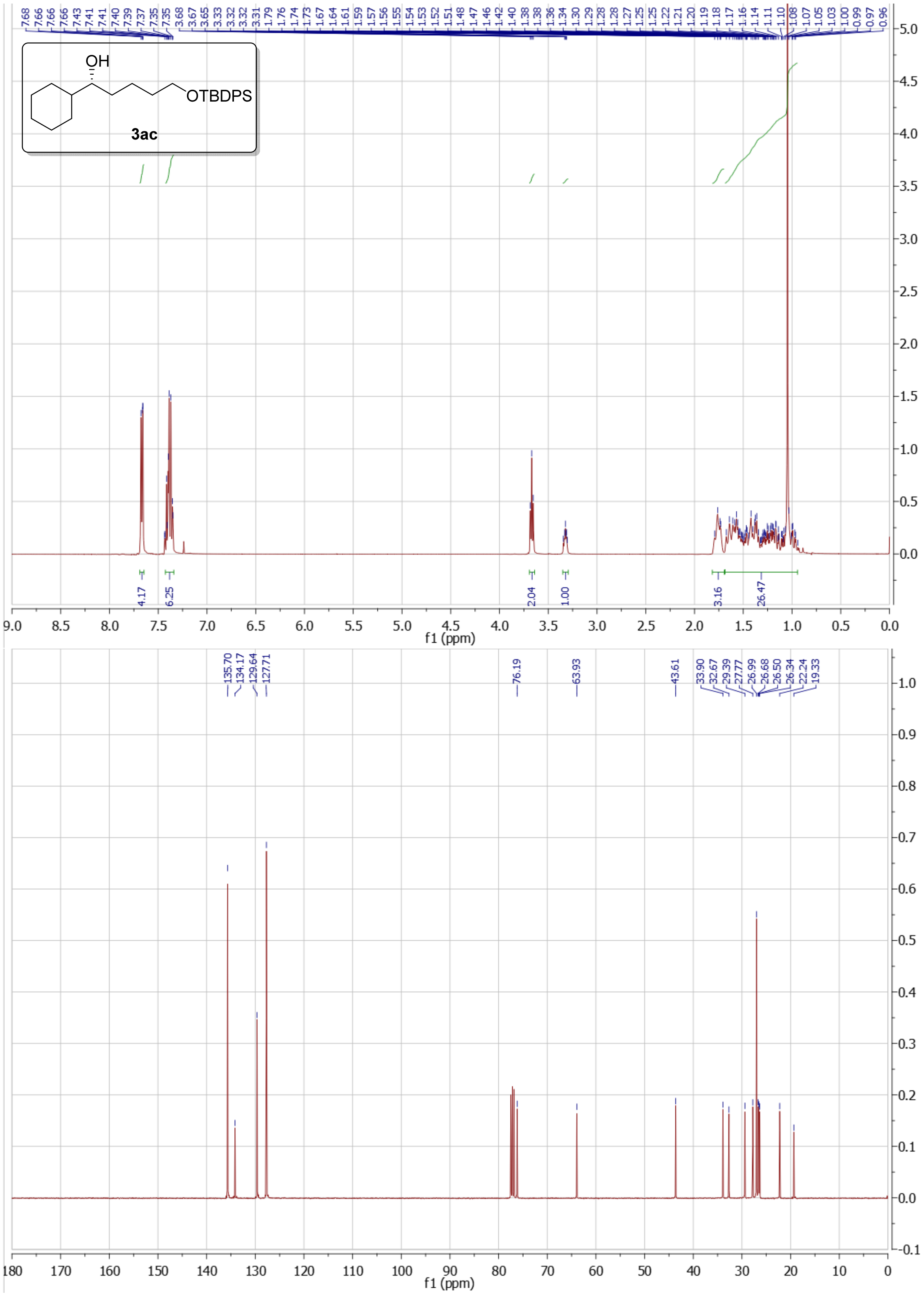

(*R*)-5-bromo-1-cyclohexylpentan-1-ol (3ad):

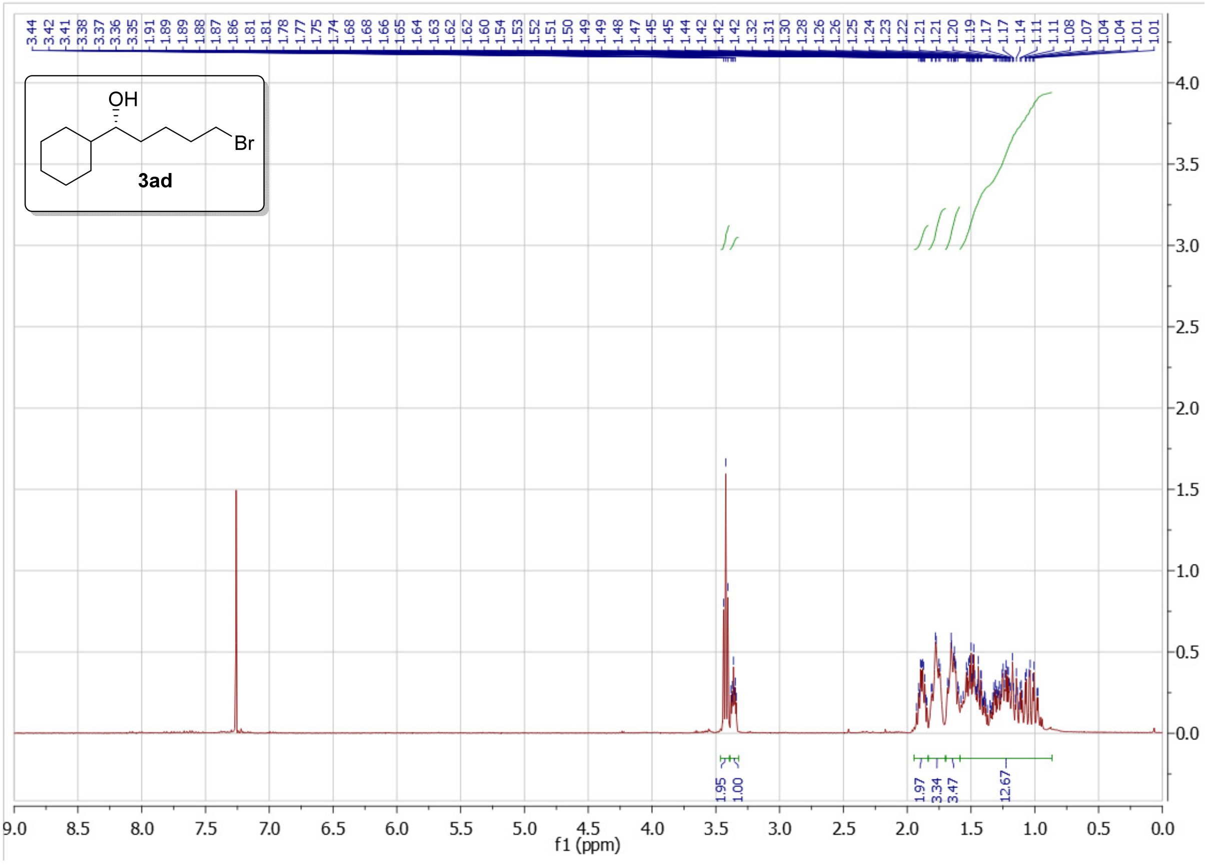

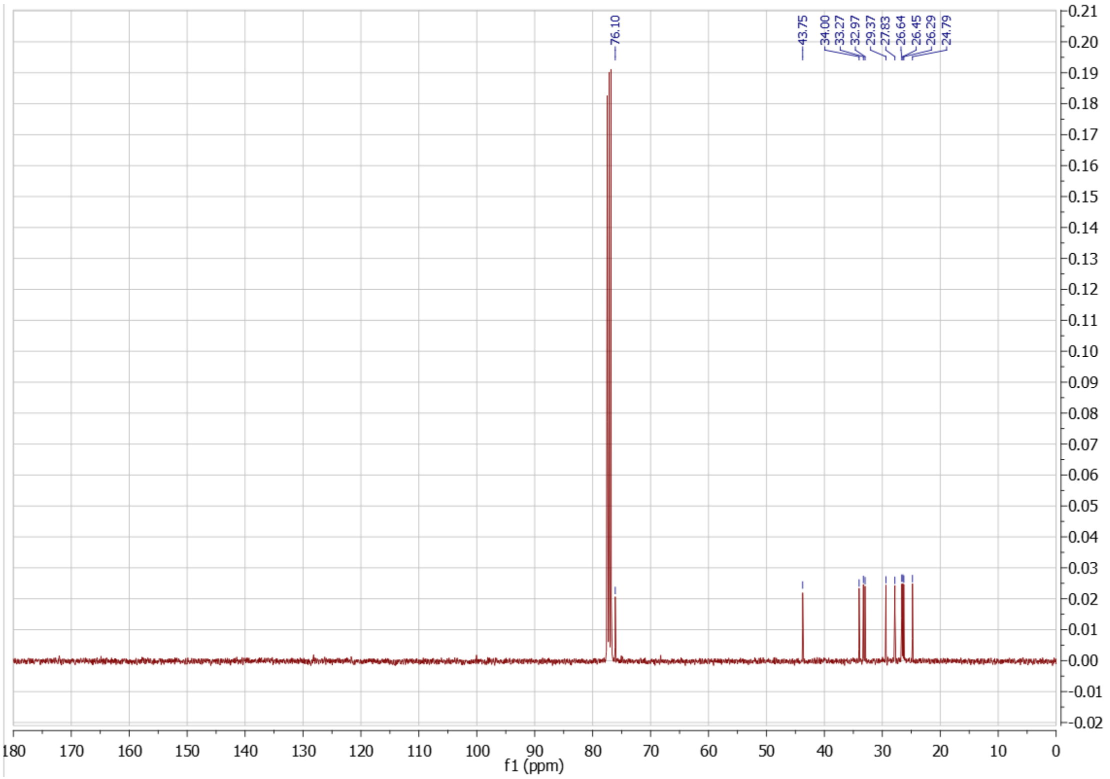

(*R*)-5-chloro-1-cyclohexylpentan-1-ol (3ae):

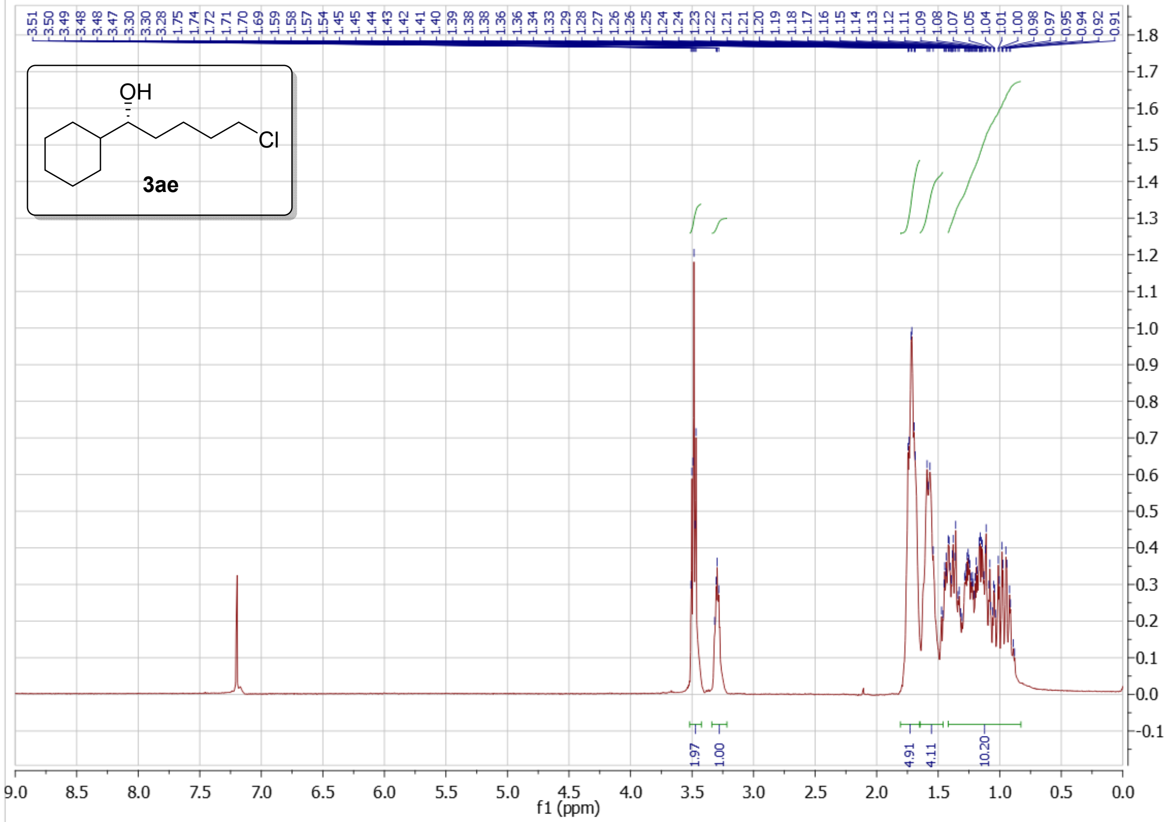

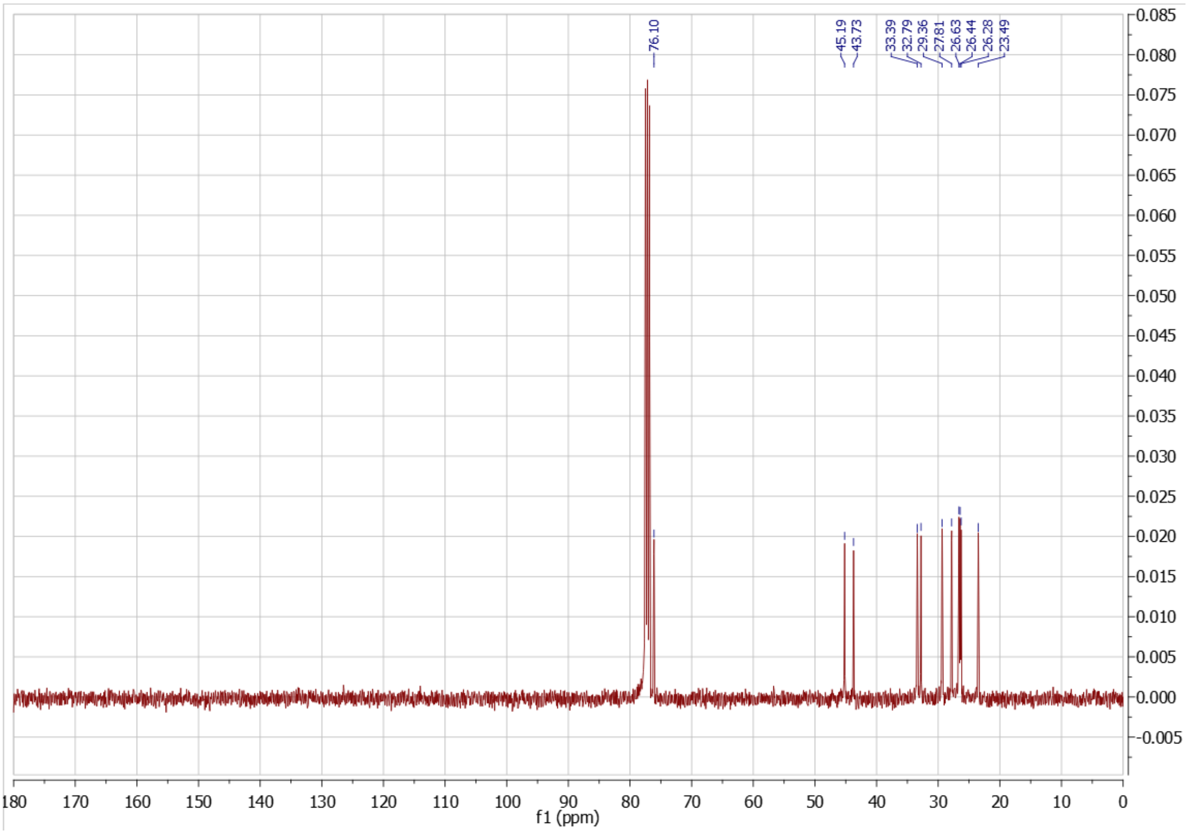

(*R*)-tetradecan-7-yl benzoate (3ea′):

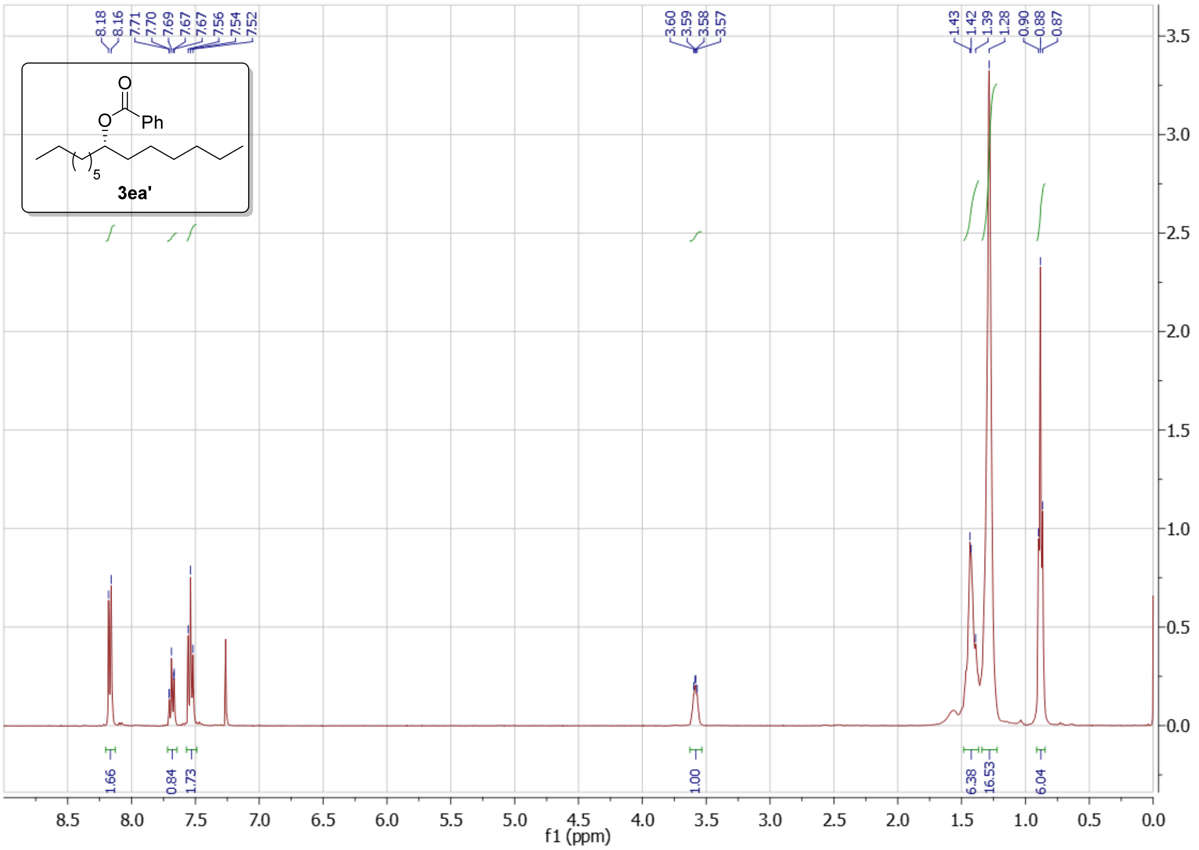

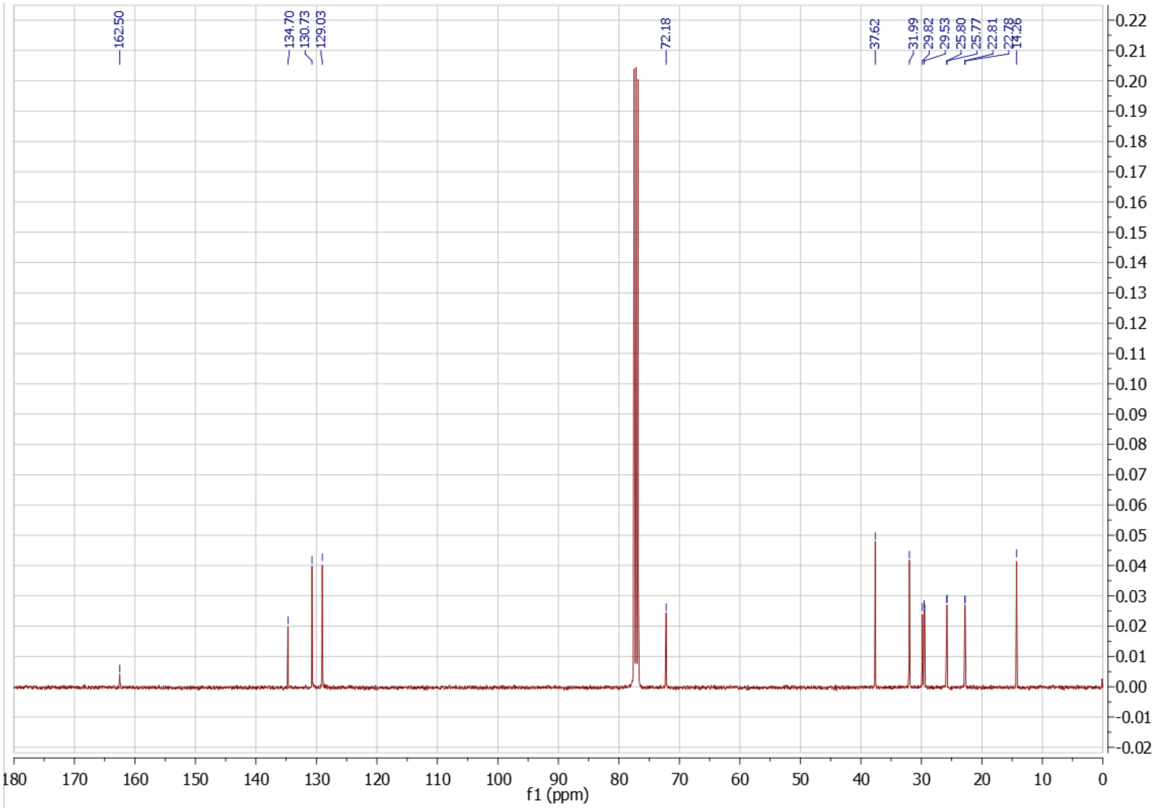

(*R*)-5-bromo-1-cyclohexylpentyl benzoate (3ad′):

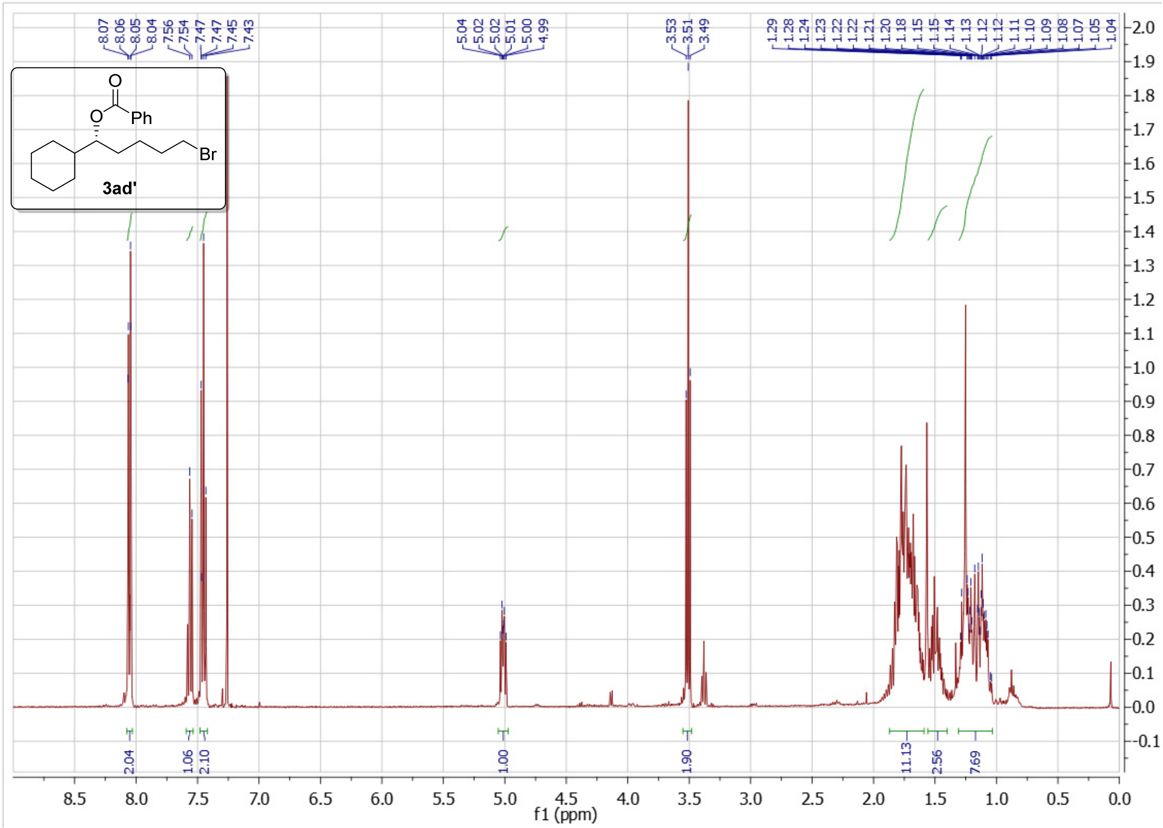

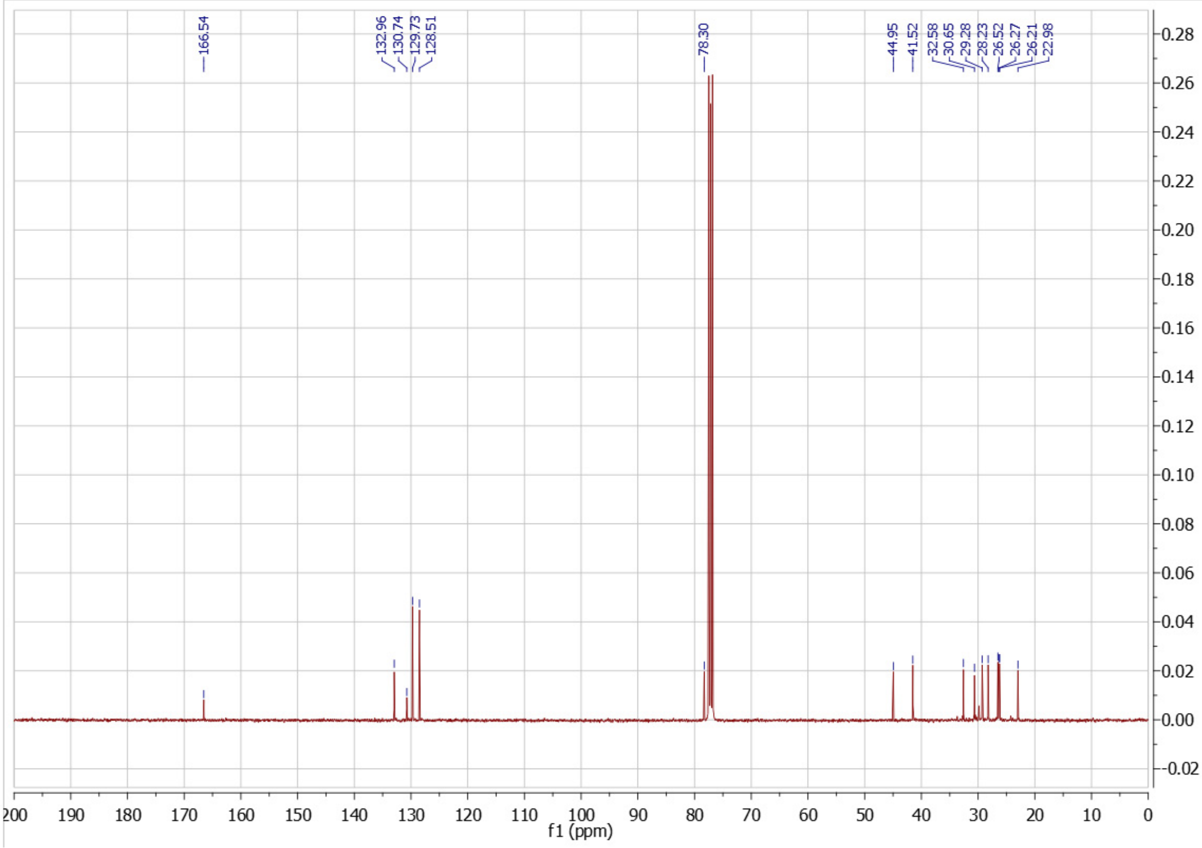

(*R*)-5-chloro-1-cyclohexylpentyl benzoate (3ae′):

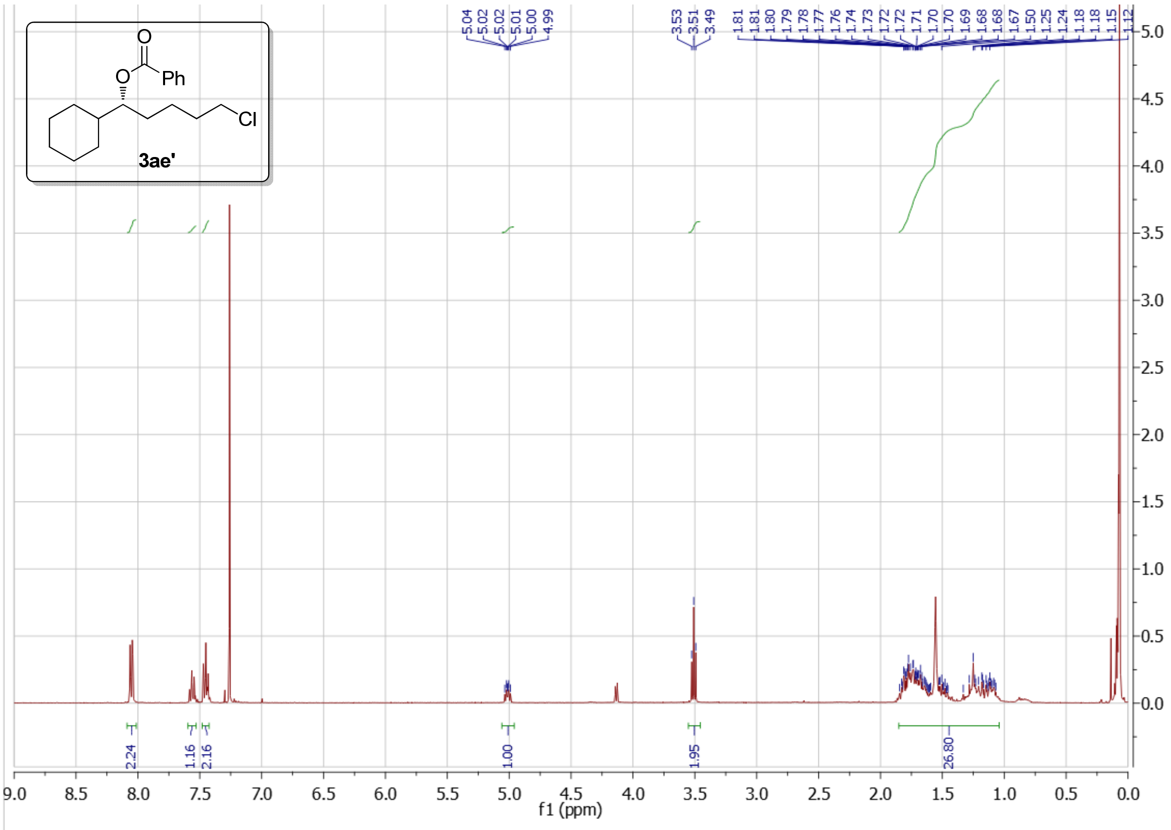

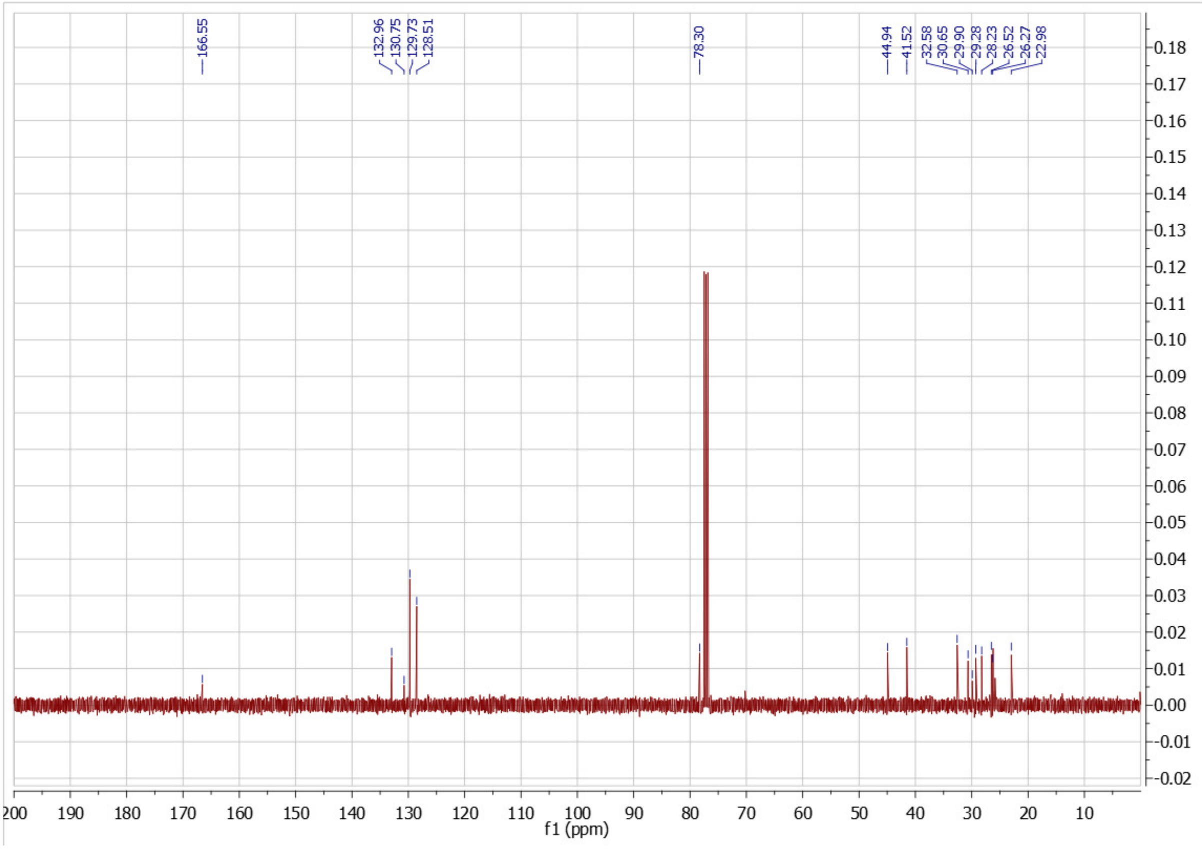

IR spectra for new compounds

(*R*)-3-ethylnonan-4-ol (3ca):

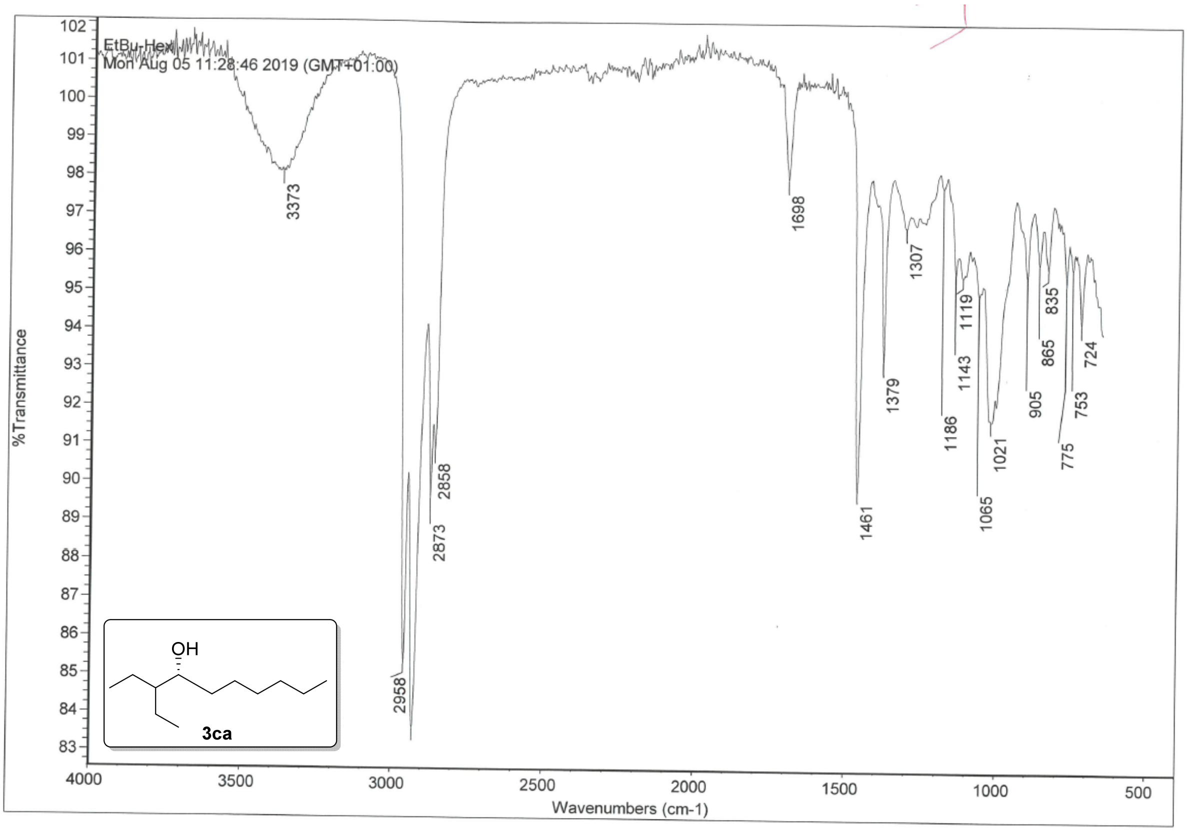

(*R*)-tetradecan-7-ol (3ea):

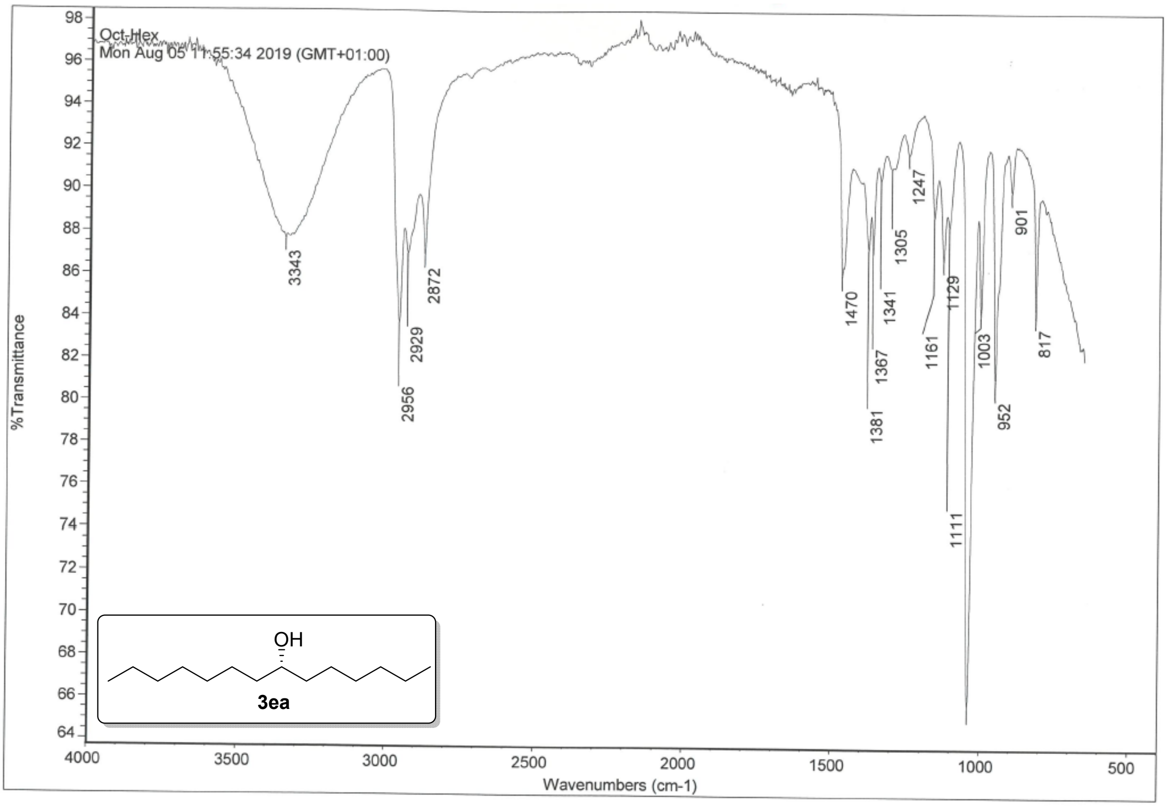

(*R*)-1-cyclohexyl-6-phenylhexan-1-ol (3ab):

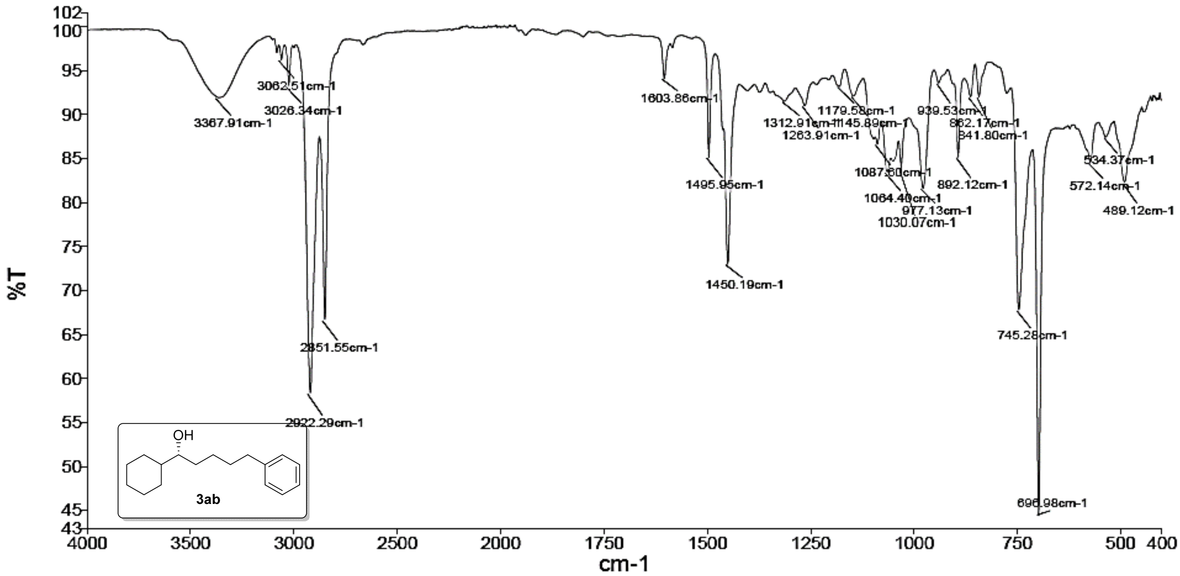

(*R*)-5-(*tert*-butyl-dimethyl-silanyloxy)-1-cyclohexylheptan-1-ol (3ac):

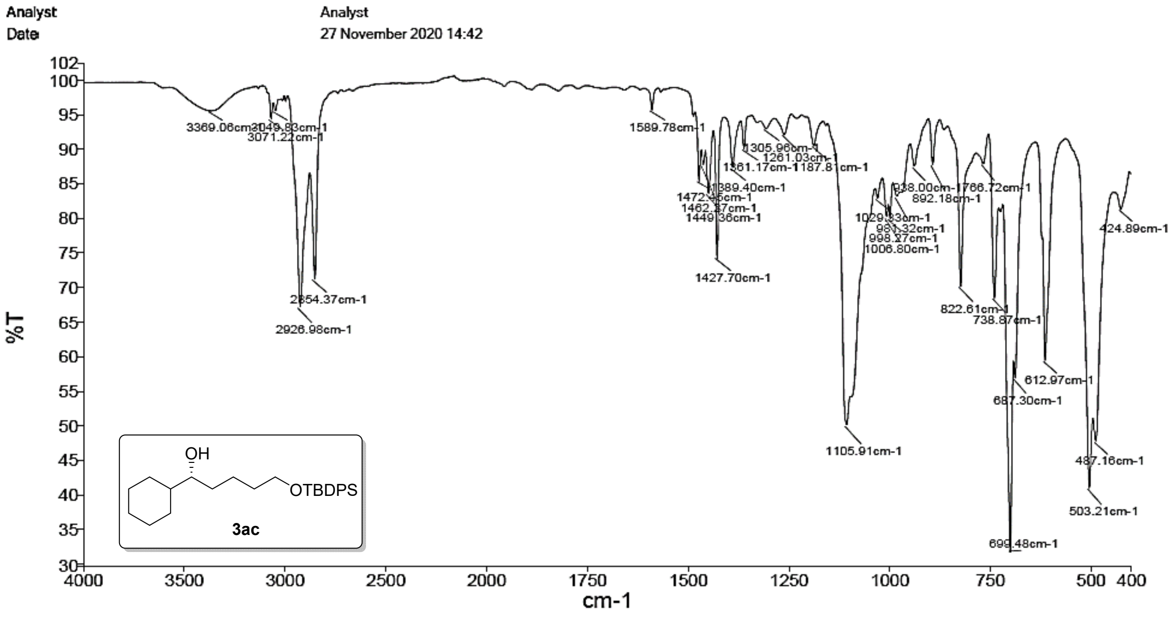

(*R*)-tetradecan-7-yl benzoate (3ae′):

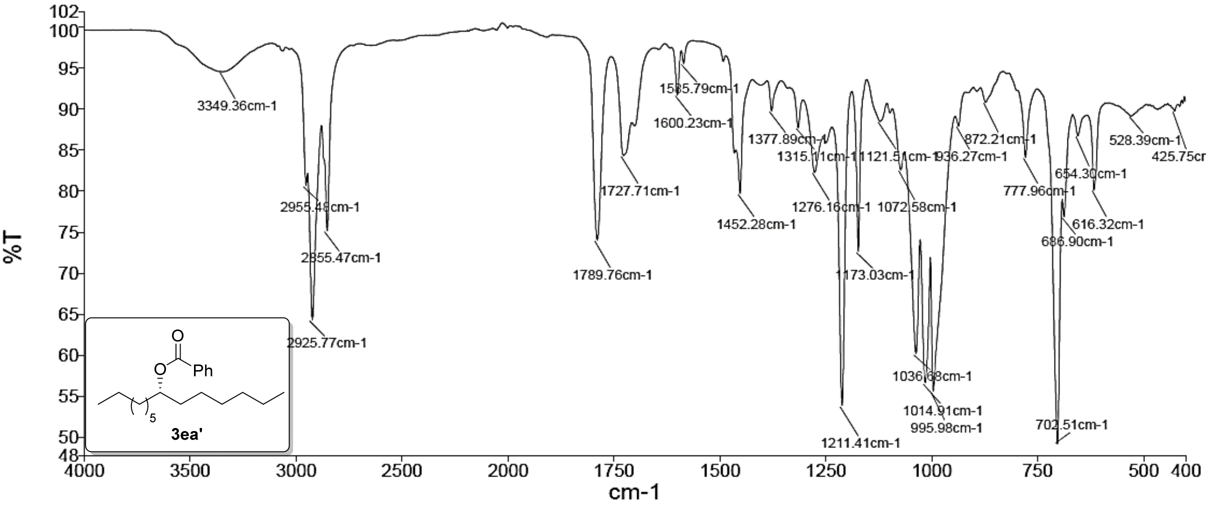

(*R*)-5-bromo-1-cyclohexylpentyl benzoate (3ad′):

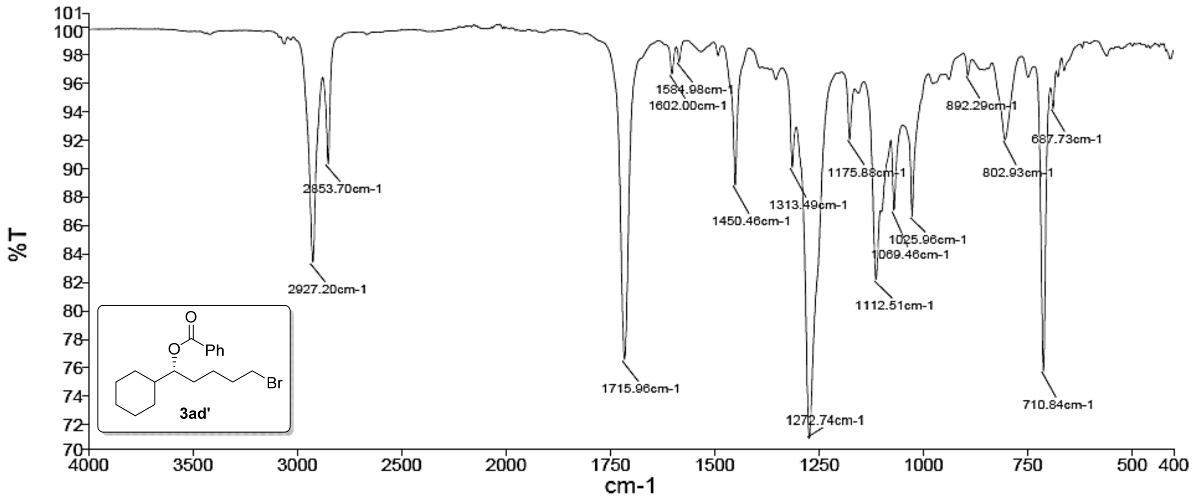

(*R*)-5-chloro-1-cyclohexylpentyl benzoate (3ae′):

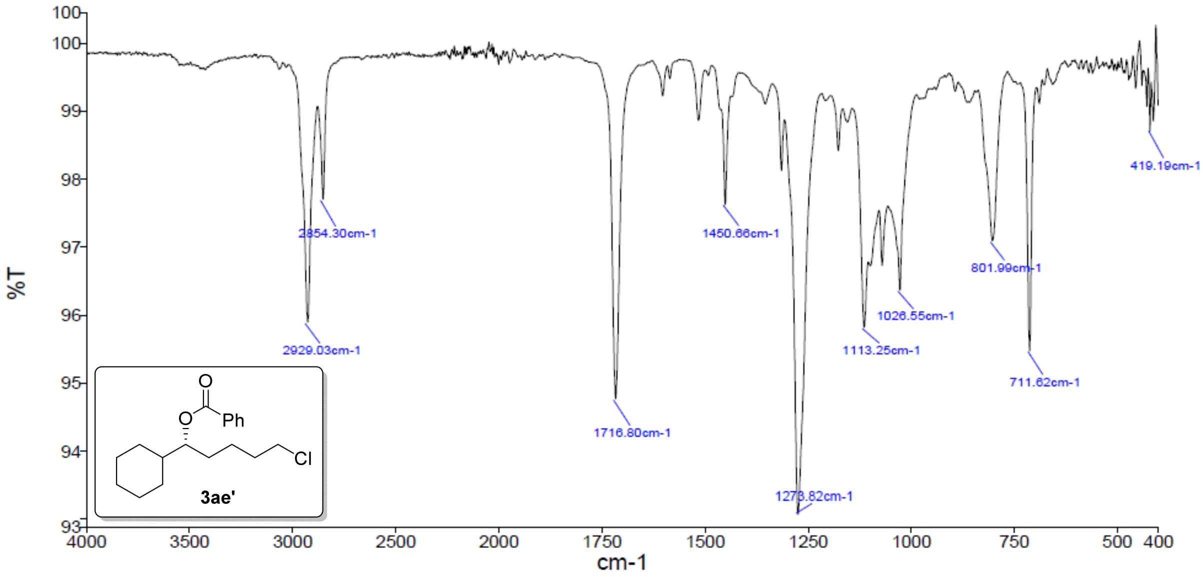

Supplement: Supplementary file 1 [file molecules-26-04471-s001.zip › molecules-1298950-supplementary.pdf]
